# Evolutionary and developmental dynamics of sex-biased gene expression in common frogs with proto-Y chromosomes

**DOI:** 10.1186/s13059-018-1548-4

**Published:** 2018-10-05

**Authors:** Wen-Juan Ma, Paris Veltsos, Roberto Sermier, Darren J Parker, Nicolas Perrin

**Affiliations:** 1 Department of Ecology and Evolution, University of Lausanne, CH 1015 Lausanne, Switzerland; 2 Current address: Department of Biology, Amherst College, Amherst, MA USA; 3 Swiss Institute of Bioinformatics, Lausanne, Switzerland

**Keywords:** Sex bias, Gene expression, Evolutionary rate, Sexually antagonistic genes, Development, Sex reversals, Proto-sex chromosome, Sexualization, Transcriptional degeneration, Faster-X effect

## Abstract

**Background:**

The patterns of gene expression on highly differentiated sex chromosomes differ drastically from those on autosomes, due to sex-specific patterns of selection and inheritance. As a result, X chromosomes are often enriched in female-biased genes (feminization) and Z chromosomes in male-biased genes (masculinization). However, it is not known how quickly sexualization of gene expression and transcriptional degeneration evolve after sex-chromosome formation. Furthermore, little is known about how sex-biased gene expression varies throughout development.

**Results:**

We sample a population of common frogs (*Rana temporaria*) with limited sex-chromosome differentiation (proto-sex chromosome), leaky genetic sex determination evidenced by the occurrence of XX males, and delayed gonadal development, meaning that XY individuals may first develop ovaries before switching to testes. Using high-throughput RNA sequencing, we investigate the dynamics of gene expression throughout development, spanning from early embryo to froglet stages. Our results show that sex-biased expression affects different genes at different developmental stages and increases during development, reaching highest levels in XX female froglets. Additionally, sex-biased gene expression depends on phenotypic, rather than genotypic sex, with similar expression in XX and XY males; correlates with gene evolutionary rates; and is not localized to the proto-sex chromosome nor near the candidate sex-determining gene *Dmrt1*.

**Conclusions:**

The proto-sex chromosome of common frogs does not show evidence of sexualization of gene expression, nor evidence for a faster rate of evolution. This challenges the notion that sexually antagonistic genes play a central role in the initial stages of sex-chromosome evolution.

**Electronic supplementary material:**

The online version of this article (10.1186/s13059-018-1548-4) contains supplementary material, which is available to authorized users.

## Background

Sexual dimorphism is a nearly universal feature of species with separate sexes. Phenotypic differences between the sexes are assumed to reflect past or ongoing sexual conflicts: trait values that facilitate gene transmission through the male function might impede gene transmission through the female function [1]. Hence, the phenotypic trait values that maximize male fitness might often differ from those that maximize female fitness. Although the genetic bases underlying sexually dimorphic traits are often complex and polygenic [2], they fall into two broad categories, reflecting two alternative ways of solving sexual conflicts. On one hand, sexual dimorphism may arise from the differential expression of autosomal genes, via, e.g., hormonal control [3–5]. This is the only option available to species with non-genetic sex determination. On the other hand, species with genetic sex determination potentially benefit from an alternative option based on sex-chromosome differentiation: as Y chromosomes only occur in males, they may safely accumulate sexually antagonistic male-beneficial alleles without jeopardizing female fitness. Reciprocally, X chromosomes spend two thirds of their time in females, which selects for female-beneficial alleles (though in the case of differentiated sex chromosome with silenced Y copies, male-beneficial alleles might segregate on X chromosomes if recessive) [6]. The same holds for female-heterogametic systems, in which W chromosomes are female limited, while Z chromosomes spend two thirds of their time in males and hence are expected to accumulate male-beneficial genes.

Sexually antagonistic genes are hypothesized to play a key role in the evolution of sex chromosomes. In proto-sex chromosomes (where X and Y chromosomes differ only at the sex-determining locus), male-beneficial mutations on the Y may spread even if detrimental to females, because linkage with the sex-determining locus makes them more likely to be transmitted to sons than to daughters. These might be mutations affecting coding sequences or promoter regions, but also DNA methylation or heterochromatinization affecting transcriptional activity, such that alleles from one gametolog (e.g., X) might be upregulated, and those of the other gametolog downregulated. In turn, the accumulation of sexually antagonistic alleles is expected to select for an arrest of XY recombination meaning male-beneficial alleles will then be only transmitted to sons (and female-beneficial alleles to daughters), thereby suppressing recombination load [7]. As a side consequence, however, recombination arrest will also trigger the accumulation of deleterious mutations on the Y chromosome due to reduced purifying selection and increased strength of genetic drift stemming from their low effective population size (approximately one fourth that of autosomes). Over time, loss-of-function mutations may accumulate in Y-linked genes, resulting in the degeneration of non-recombining segments of Y chromosomes [8–10]. Thus, while the sex-biased expression of autosomal genes is thought to result from sexual conflict alone, sex-biased expression of sex-linked genes potentially arises from a combination of sexualization and decay [11].

So far, the sex-biased expression of autosomal and sex-linked genes has mostly been investigated in species with highly differentiated sex chromosomes, using model organisms such as mammals, birds, or insects [4, 12–16], and with a focus on adult tissues (so that little is known about the dynamics of sex-biased gene expression throughout development) [3, 17, 18]. One general outcome of such studies is that X chromosomes are often enriched in female-biased genes (i.e., feminized) and Z chromosomes in male-biased genes (i.e., masculinized), as expected from their preferential occurrence in females and males respectively (reviewed in [19]). Another common pattern in adult gonad tissues shown by sex-biased genes, regardless of their genomic locations, is that more genes are biased towards male expression than towards female expression [3, 20–23]. Furthermore, male-biased genes consistently show greater between-species divergence than female-biased and unbiased genes, at both gene expression and coding sequence levels [3, 17]. These patterns suggest that the evolution of sex-biased genes is largely driven by selection on males, most likely stemming from sexual selection and sexual conflict, which are typically stronger in males [21, 24]. In addition, sex-linked genes also often show a rapid evolutionary rate (so-called faster-X or faster-Z effect), which likely stems from both the lower effective population size of sex chromosomes and the exposure to selection of hemizygous genes in the heterogametic sex.

It is not clear, however, how fast gene expression of sex-linked genes becomes sexualized (e.g., feminization of X or masculinization of Z chromosomes) and how quickly signatures of selection can be detected following the birth of sex chromosomes [25–27]. This requires the study of gene expression and coding sequence diversity from sex chromosomes at multiple differentiation stages. In this context, the European common frog (*Rana temporaria*) is an ideal species, because it is polymorphic for sex-chromosome differentiation [28]. At one extreme are populations, found at high latitudes or altitudes, with differentiated X and Y chromosomes (evidenced by Y-specific alleles fixed at series of genetic markers along the whole sex chromosome genetic map), associated with strictly genetic sex determination (GSD) [28, 29]. At the other extreme are populations, found under mild climatic conditions, with undifferentiated XX chromosomes and non-genetic sex determination (non-GSD) [30]. Populations at intermediate climatic conditions contain a mix of XY males (with differentiated sex chromosomes), XX males (with undifferentiated sex chromosomes, genetically similar to XX females), and/or XY° males (with proto-Y chromosomes, only differentiated at a small genomic region around the candidate sex-determining gene *Dmrt1*) [31–33], together with rare sex-reversed XY or XY° females [32, 33]. Sex determination in these populations is under partial genetic control (“leaky GSD”): XX individuals tend to develop into females, but also have a significant probability of developing into sex-reversed XX males; XY individuals most often develop as males, but also have a low probability of developing into sex-reversed XY females. Sex-chromosome recombination in these rare XY females produces XY° sons with proto-sex chromosomes [31, 34].

This polymorphism in the patterns of sex-chromosome differentiation seemingly fits the concept of “sex races,” described from common frogs in the 1930s based on the patterns of gonadal development [35, 36]. Juveniles from the “differentiated sex race” display early and direct gonadal differentiation: juveniles at metamorphosis (Gosner stage 43 [37]) all present either testes or ovaries in balanced numbers, in association with strict GSD. Those from the “undifferentiated sex race” display delayed and indirect gonadal development: all juveniles present ovaries at metamorphosis, and only later in development (mostly before Gosner stage 46) do some of them replace ovaries by testes. In between, populations from the “semi-differentiated sex race” present an intermediate situation: a majority of juveniles have ovaries at metamorphosis, but a few already have testes, and some others an intermediate condition (ongoing transition from ovaries to testes) [35, 36].

Here we focus on one such population from the semi-differentiated sex race comprising a majority of XY° males together with a few sex-reversed XX males. Through RNAseq analyses of different families and developmental stages, we ask the following questions: (i) Do sex chromosomes at an early stage of evolution show signs of differentiation, such as altered expression of Y gametologs or signatures of increased selection (i.e., faster-X effect)? (ii) Has transcriptional sexualization already started, i.e., are proto-sex chromosomes already enriched in sex-biased genes compared to autosomes? (iii) Does sex bias in gene expression (whether sex-linked or autosomal) depend on genotypic or on phenotypic sex, and do sex-biased genes display faster rates of evolution? (iv) How does sex bias change along developmental stages, and in particular, do these patterns reflect the complex developmental pathways documented from the semi-differentiated sex race (namely, direct versus indirect development of testes)?

## Results

### Genotypic and phenotypic sexes

Analysis of field-sampled adults with *Dmrt* markers and sex-linked microsatellites revealed that all 24 females were XX, 26 males out of 28 were XY°, and two were XX (Additional file 1). The population under study can therefore be assigned to the semi-differentiated sex race, with a majority of males presenting proto-sex chromosomes and a small proportion of sex-reversed XX males. The parents of the six collected families comprised six XY° fathers and six XX mothers. *Dmrt* analysis of their progeny sampled for RNAseq analyses revealed three to seven XX and XY° individuals respectively at each stage, for a total of 46 samples. The phenotypic sexing of stages G43 and G46 established a reasonably good, but (as expected) imperfect correlation between phenotypic and genotypic sex: five XY° individuals still had ovaries at stage G43 (being expected to develop testes at a later stage) and one XX individual had testes at stage G46 (being thus expected to develop as a functional sex-reversed XX male). In the following analyses, we will contrast the gene expression of XX versus XY° individuals at the three early stages (as phenotypic sexes are undefined), while for later stages (G43 and G46), we will compare gene expression in reference to both genotypic and phenotypic sex (namely XX females, XY° males, XY° with ovaries, and XX with testes).

### Transcriptome sequencing and assembly

A total of 558,745 transcripts were assembled, of which 272,330 corresponded to unique genes, the others being splicing variants. De novo transcriptome assemblies typically consist of more contigs than can possibly be considered real, even when alternative splicing is taken into account [14]. After quality control to exclude transcripts with low expression or that had high similarity to other transcripts, a reference transcriptome containing 67,288 transcripts was produced for use in the expression analyses. BUSCO v2 [38] identified ~ 80% complete and < 3% fragmented single-copy tetrapod orthologs (*n* = 3950, C: 79.8% [S: 78.6%, D: 1.2%], F: 2.6%, M: 17.6%). Approximately 85.7% of the trimmed reads could be mapped to the reference transcriptome using Bowtie2 v2.3.1 [39].

### Sex-biased gene expression throughout development

**Fig. 1:**
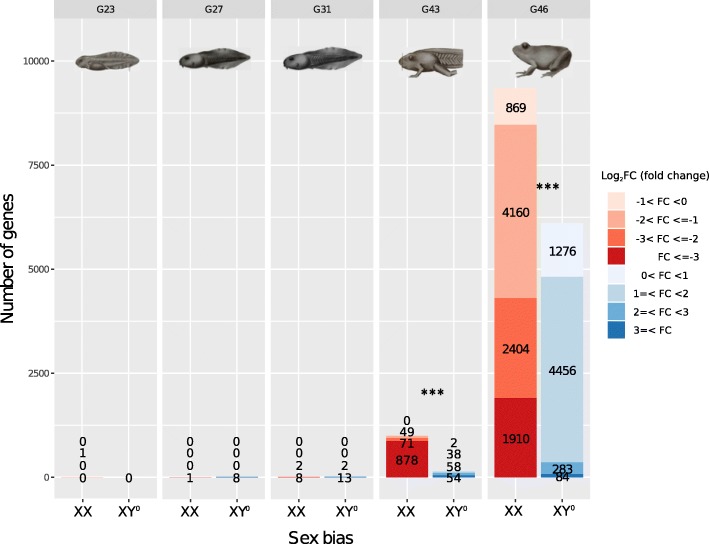
Sex bias in gene expression across developmental stages in *Rana temporaria*. The number of genes with significant sex bias (corrected for multiple testing) increases drastically in the late developmental stages (G43 and G46), corresponding to the morphological differentiation of gonads. At these stages, female-biased genes (reddish) significantly outnumber male-biased genes (blueish), mostly for the highly biased categories (|log_2_FC| ≥ 2 and ≥ 3). Drawings of frog tadpoles and larvae are reprinted from [80], with permission of the editors

After multiple-test correction (FDR = 0.05), 16,246 transcripts (24%) were significantly sex-biased in expression in at least one of the five developmental stages, of which 14,480 (21.5% of total) also had a |log_2_FC| (absolute value of log_2_ fold change difference) ≥ 1 (Table 1). The extent of sex bias increased drastically throughout development (Fig. 1). At early stages (G23 to G31), very few genes were sex biased, with no significant differences between the numbers of female- and male-biased genes (Table 1). One transcript had sex-biased expression at stage G23 (undifferentiated stage), eight at stage G27 (corresponding to the initiation of gonad development [40]), and 25 at stage G31 (when gonad differentiation becomes identifiable histologically [40, 41]). Sex bias increased strongly at the metamorph stage G43 (1148 genes with a ≥ 2-fold difference between XY° males and XX females) and even more so at the froglet stage G46 (13,297 genes with a ≥ 2-fold difference). At stages G43 and G46, many more genes were female biased (higher expression in XX females) than male biased (higher expression in XY° males), particularly those with stronger bias (|log_2_FC| ≥ 2 and ≥ 3; Fig. 1). There was little overlap between stages in the identity of sex-biased genes, with few differences from random expectation (SuperExactTest, *p* > 0.1 in most cases; Additional file 2), suggesting a rapid turnover among stages. No single gene was sex-biased across all five stages, and only 3.4% of XX-biased genes (323 out of 9680) and 1.4% of XY°-biased genes (88 out of 6217) were shared between at least two developmental stages (|log_2_FC| ≥ 1, Additional file 3: Figure S1a, b), most of which were between stages G43 and G46. Still, 79.1% of sex-biased genes identified at stage G46 (11,959 out of 15,125) were unbiased at stage G43 (|log_2_FC| ≥ 1, Additional file 3: Figure S1c).

**Table 1:** Different fold change cutoff threshold of sex-biased gene expression along five developmental stages in *Rana temporaria*

Developmental stage	Cutoff threshold (fold change)	Female-biased (%)^f^	Male-biased	Sex-biased (*p* value)^e^
Gosner stage 23	5% FDR^a^	1	0	NA
	≥ 2^b^	1	0	NA
	≥ 4^c^	0	0	NA
	≥ 8^d^	0	0	NA
Gosner stage 27	5% FDR	1 (11.1)	8	XY° bias tendency ^.^
	≥ 2	1 (11.1)	8	XY° bias tendency ^.^
	≥ 4	1 (11.1)	8	XY° bias tendency ^.^
	≥ 8	1 (11.1)	8	XY° bias tendency ^.^
Gosner stage 31	5% FDR	10 (40)	15	XY° bias tendency ^.^
	≥ 2	10 (40)	15	XY° bias tendency ^.^
	≥ 4	10 (40)	15	XY° bias tendency ^.^
	≥ 8	8 (38.1)	13	XY° bias tendency ^.^
Gosner stage 43	5% FDR	998 (86.8)	152	XX bias***
	≥ 2	998 (86.9)	150	XX bias***
	≥ 4	949 (89.4)	112	XX bias***
	≥ 8	878 (94.2)	54	XX bias***
Gosner stage 46	5% FDR	9337 (60.5)	5999	XX bias***
	≥ 2	8474 (63.7)	4823	XX bias***
	≥ 4	4314 (92.2)	367	XX bias***
	≥ 8	1910 (95.8)	84	XX bias***

^a^Based on FDR correction for multiple testing. XY°-biased: log_2_(m/f) > 0, XX-biased: log_2_(m/f) < 0

^b^XY°-biased: log_2_(m/f) ≥ 1, XX-biased: log_2_(m/f) ≤ − 1

^c^XY°-biased: log_2_(m/f) ≥ 2, XX-biased: log_2_(m/f) ≤ − 2

^d^XY°-biased: log_2_(m/f) ≥ 3, XX-biased: log_2_(m/f) ≤ − 3

^e^Significance codes are 0.001 ‘***’, 0.1 ‘^.^’

^f^Percentage among all sex-biased genes at certain developmental stage within a certain fold change category

### Genomic locations of sex-biased genes

Based on the strong genome-wide synteny between *R. temporaria* and *Xenopus tropicalis* [42, 43], we performed a reciprocal best BLAST of coding sequences between these two species to identify the genomic locations of orthologs (see details in the “Methods” section). A total of 10,756 *X. tropicalis* orthologs could be identified with one-to-one reciprocal best BLAST hit, with no significant bias among chromosomes (except for a slight deficit on chromosome 9; Additional file 4: Table S1). Among these were 20.0% of the genes upregulated in XY° males (993 out of 4973) versus 40.8% of the genes upregulated in XX females (3856 out of 9472) in at least one developmental stage (FDR < 0.05), a highly significant difference (*χ*^2^ = 330.0, *p* < 2.2e−16). As only one ortholog could be detected among the genes that were sex-biased at early stages (G23 to G31), the genomic localization of sex-biased genes was only analyzed for stages G43 and G46 (with respectively 207 and 4642 orthologs identified).

**Fig. 2:**
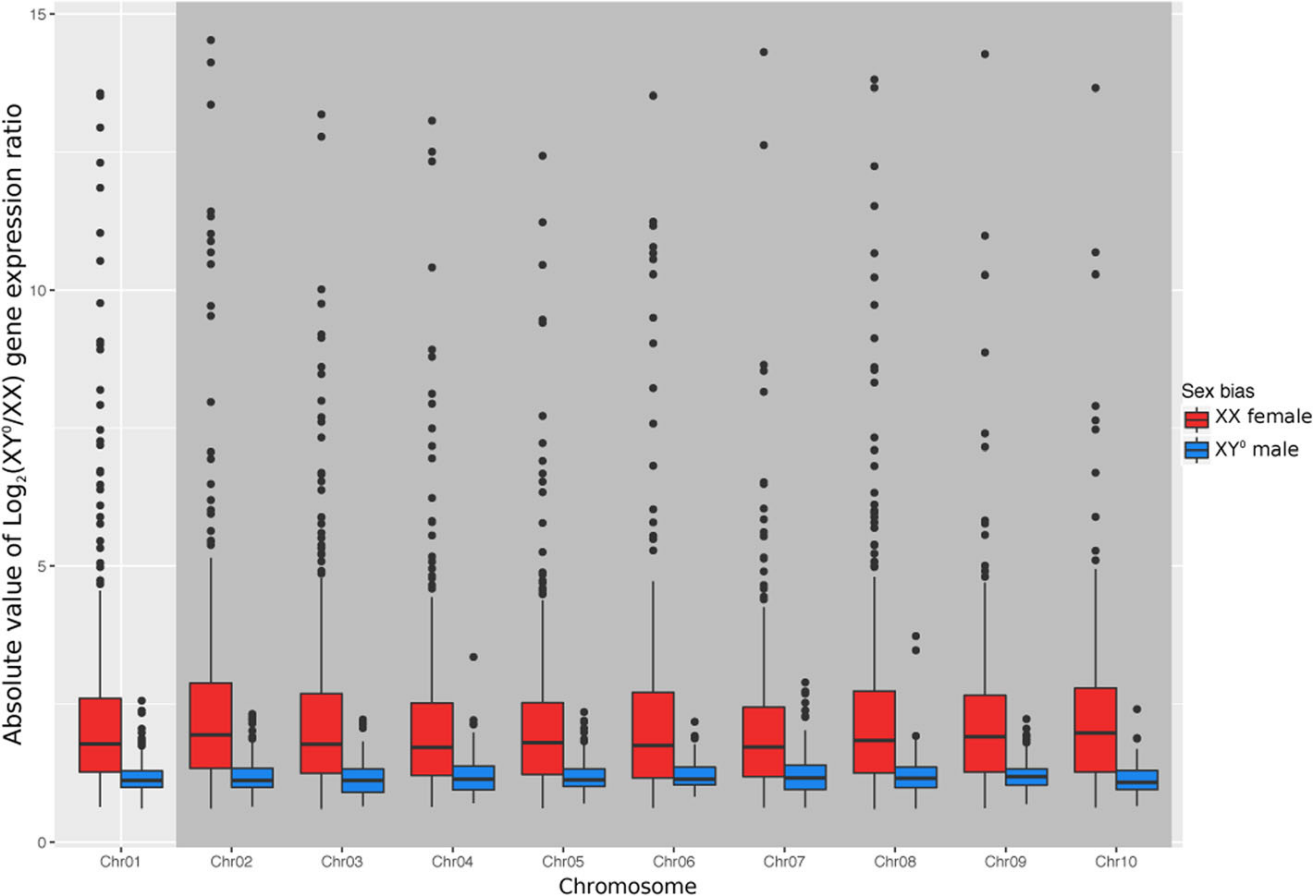
Female-biased genes (red) and male-biased genes (blue) in froglets (G46) show the same distribution patterns on the sex chromosomes (Chr01, left) as on autosomes (Chr02 to 10, right, shaded area)

The only distinctive feature of sex chromosomes was a slight deficit at stage G46 in genes biased for XY° males (among the sex-biased ones), as compared to autosomes: 17.1% (122 out of 714) of the sex-biased genes on sex chromosomes were male biased, versus 21.5% (846 out of 3928) on autosomes (*χ*^2^ = 4.66, *p* = 0.03). No such deficit occurred at stage G43, with six male-biased genes out of 25 sex-biased on sex chromosomes versus three out of 182 on autosomes (*χ*^2^ = 0.19, *p* = 0.67). On all other accounts, sex chromosomes did not differ from autosomes. (i) The proportion of sex-biased genes did not differ between sex chromosomes and autosomes, both at G43, with 2.8% (39 out of 1418 orthologs) on sex chromosomes versus 2.1% (168 out of 8047) on autosomes (*χ*^2^ test, *p* = 0.14), and at G46, with 35.3% (714 out of 2025) on sex chromosomes versus 34.6% (3928 out of 11,347) on autosomes (*χ*^2^ test, *p* = 0.54). (ii) Over all the orthologs found, the ratio of XY° male to XX female expression did not differ between sex chromosomes and autosomes, at both G43 (Wilcoxon test, *W* = 4,161,700; *p* = 0.11) and G46 stages (Wilcoxon test, *W* = 41,981,000; *p* = 0.53; Additional file 3: Figure S2a, b). Furthermore, we did not detect an increase in bias around the sex-determination region (Additional file 3: Figure S3a, b). (iii) The same result was found when the analysis was restricted to significantly sex-biased genes: the ratio of XY° male to XX female expression did not differ between sex chromosome and autosomes, both for genes upregulated in XY° males (G43: *W* = 59, *p* = 0.98; G46: *W* = 110,760, *p* = 0.54) and those upregulated in XX females (G43: *W* = 2837, *p* = 0.17; G46: *W* = 1,207,300, *p* = 0.53, Fig. 2; Additional file 3: Figure S4a, b, c), and no pattern was found either along the sex chromosome at both stages (Additional file 3: Figure S5a–d).

### Differential expression of X and Y° genes and phenotypic vs genotypic sex

**Fig. 3:**
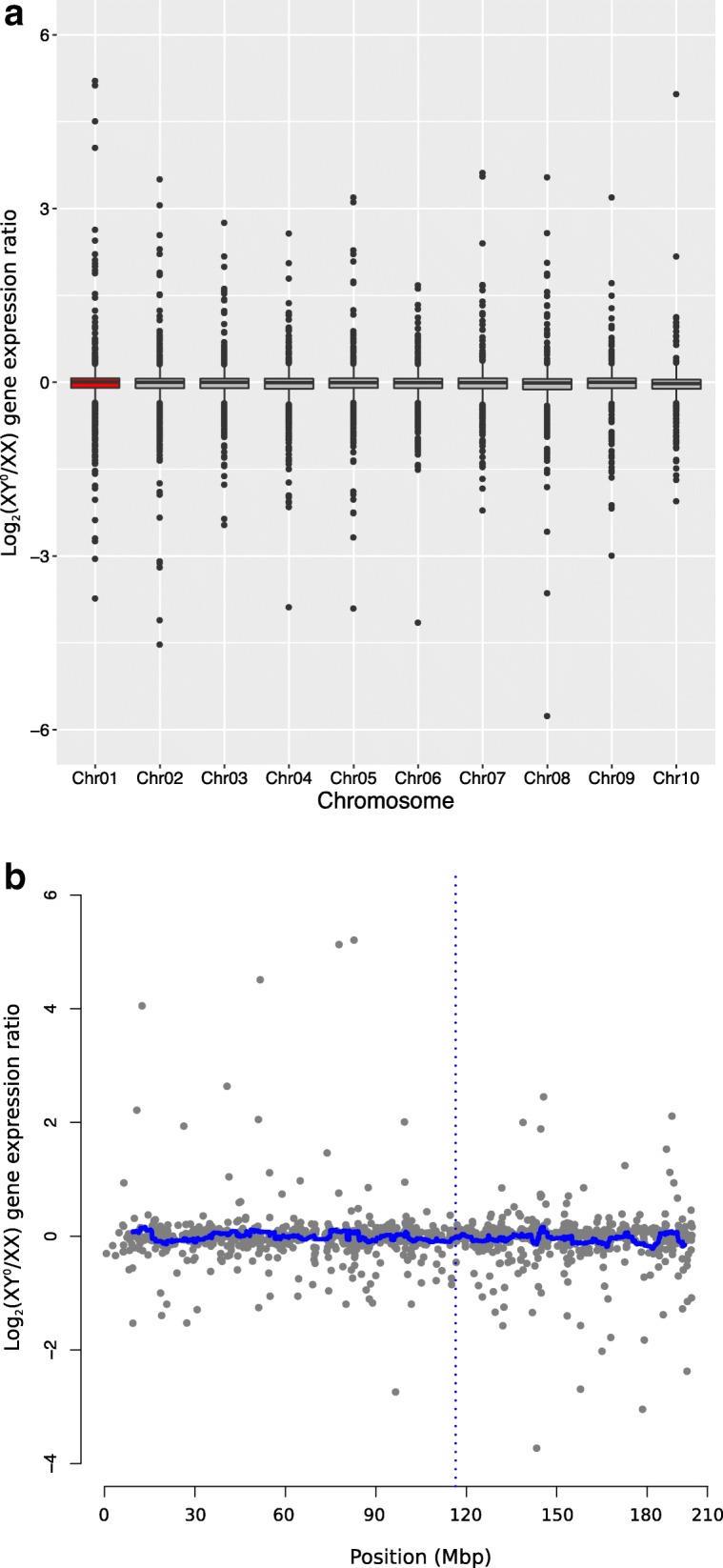
XX and XY° male froglets (G46) show similar patterns of gene expression, with **a** no specific signature of sex chromosomes (Chr01, red, left) relative to ausosomes (boxplots of Log_2_(XY°/XX) gene expression ratio) and **b** no difference around the sex-determining region (Manhattan plot of log_2_(XY°/XX) gene expression ratio along the sex chromosome, with a sliding window of 40 genes; *Dmrt1* position marked by the blue dotted line)

Our study system offers a unique opportunity to test whether sex-chromosome differentiation (XY° versus XX) affects gene expression independent of any phenotypic sex effect. To address this, we first analyzed the total gene expression profile using multiple dimensional scale analysis, which showed a grouping of XX male with XY° males, clearly separated from the XX female group (Additional file 3: Figure S6). We then compared gene expression at G46 between the XX male and either the three XY° males or the three XX females. Only 41 genes (0.06%) differed significantly in expression level between the XX male and the XY° males (two of which had an *X. tropicalis* ortholog, on chromosomes 2 and 7 respectively), as opposed to 8739 genes between this XX male and the three XX females. Furthermore, the vast majority of identified sex-biased genes in the contrast between XX females and the XX male (female bias, 6433 out of 6473; male bias, 2283 out of 2285) overlapped with the identified sex-biased genes in the contrast between XX females and XY° males (Additional file 3: Figure S7a, b). In addition, we found no difference between autosomes and sex chromosomes in the ratio of XY° to XX male expression (*W* = 5,163,700; *p* = 0.10), and this ratio did not vary along the sex chromosome (Fig. 3a, b). Overall, we found no evidence for a differential gene expression between X and Y° chromosomes.

**Fig. 4:**
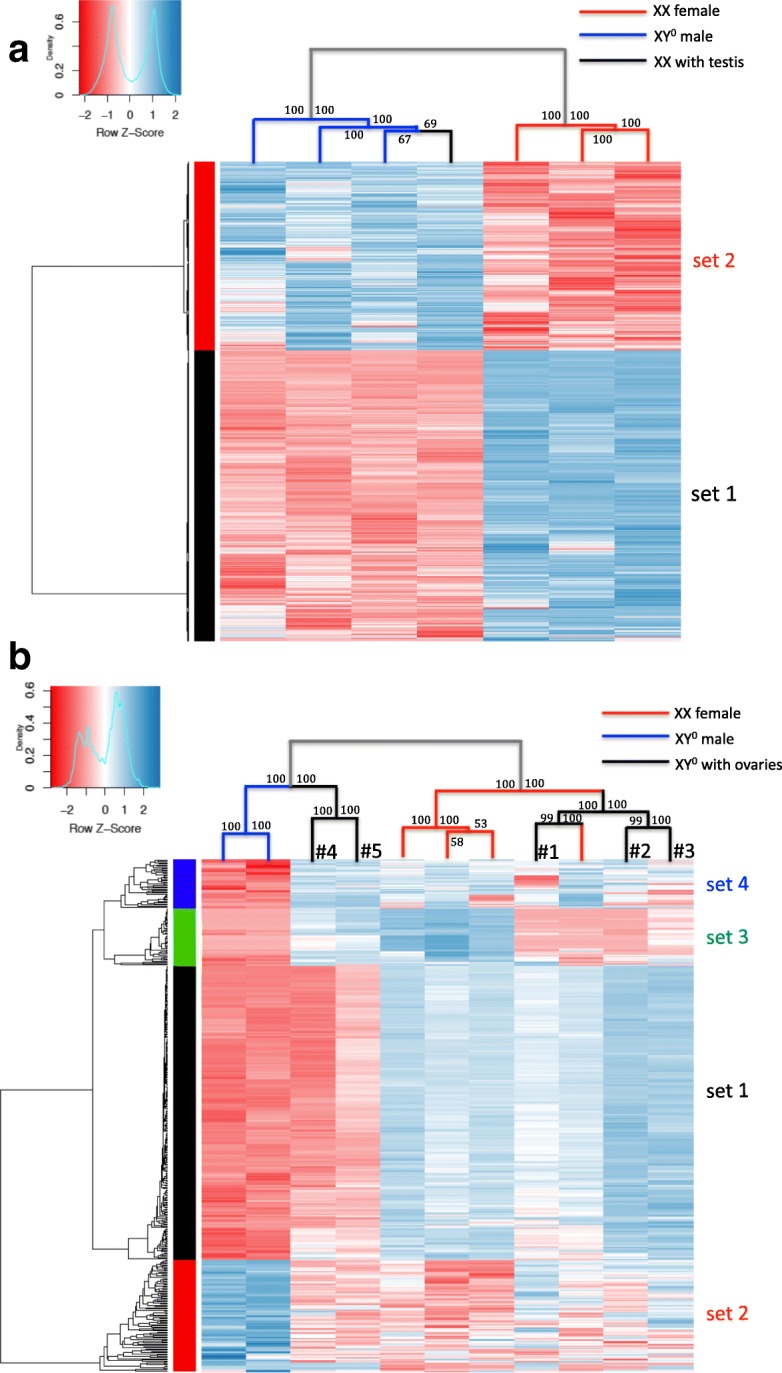
Heatmaps and hierarchical clustering of differentially expressed genes (FDR < 0.05) for XX females, XY° males, XX with testes, and XY° with ovaries at stages G46 (**a**) and G43 (**b**). Blue and red colors represent high and low expression, respectively. On each node of the clustering tree, bootstrap support values are shown from 10,000 replicates

This finding was consistent with heatmap and hierarchical clustering analysis performed on differentially expressed genes (FDR < 0.05) of these seven G46 individuals. Individuals were contrasted either by phenotypic sex (four males vs three females; Fig. 4a) or by genotypic sex (three XY° vs four XX; Additional file 3: Figure S8). In both cases, individuals cluster into the same two well-separated groups (i.e., independent of the imposed partitioning), comprising respectively the four phenotypic males and the three phenotypic females. In both cases, sex-biased genes also cluster into two well-separated sets: a larger one (set 1) comprising genes upregulated in phenotypic females and a smaller one (set 2) with genes upregulated in phenotypic males. Hence, the patterns of gene expression clearly covary with phenotypic sex, not with genotypic sex (i.e., the XX with testes clusters with XY° males, not with XX females).

A similar analysis at stage G43 (metamorphs) provides more complex results (Fig. 4b). Eleven individuals were analyzed: two XY° males, five XY° individuals with ovaries (expected to develop later into males), and four XX individuals with ovaries (some of which might later develop as males). The two XY° males and four XX females cluster into two well-differentiated groups. By contrast, XY° individuals with ovaries fall into two categories: two of them (#4 and #5 in Fig. 4b) cluster with XY° males and three (#1, #2, and #3 in Fig. 4b) with XX females. Differentially expressed genes can be categorized into four sets: the largest one (set 1) is responsible for the differentiation between the two main clusters of individuals, being upregulated in the cluster with (normal) XX females, and downregulated in the cluster with (normal) XY° males. Two other gene sets mostly differentiate the two XY° males, being respectively upregulated (set 2) or downregulated (set 4) in these two individuals. Finally, the fourth set (set 3) shows upregulation in three of the four XX females and two XY° individuals with ovaries and downregulation in all others. Thus, XY° with ovaries form a heterogeneous category: those clustering with XY° males show the same low-level expression for gene set 1 (largest set) but are otherwise similar to XX females. The XY° with ovaries clustering with XX females have an overall female-like expression profile, except for gene set 3, where they have the same low expression profile as males. Finally, one XX individual clusters with this latter group (XY° with ovaries), possibly suggesting a future development towards a male phenotype. Gene ontology (GO) analysis of these four sets of genes (Additional file 4: Table S2) shows that set 1 is enriched in genes with reproductive and immune functions, while the other three sets of genes involve no reproduction-related function or association to specific pathways of sexual development. This complex situation might represent different developmental stages in the differentiation process of male phenotypes, and possibly distinct pathways towards maleness.

### Divergence of sex-biased and sex-linked genes

**Fig. 5:**
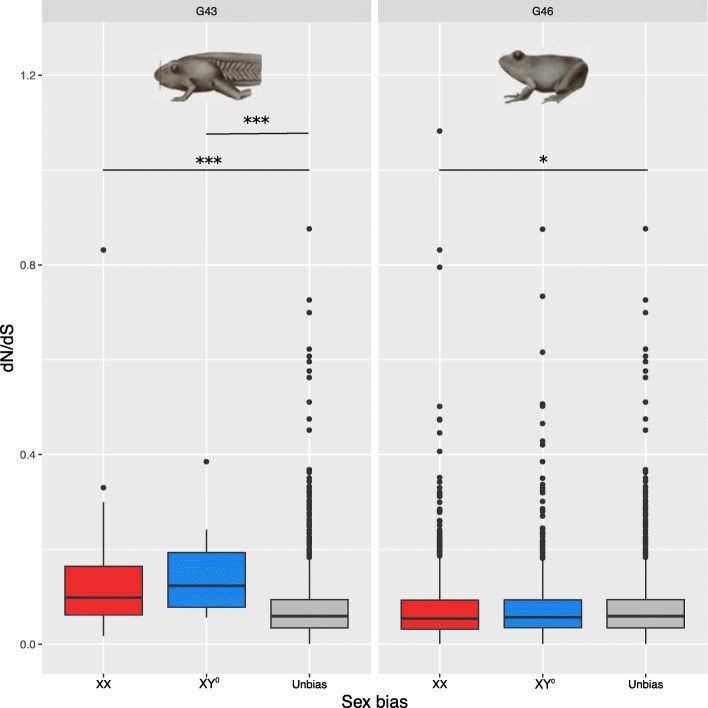
Boxplots of ratios of non-synonymous to synonymous substitutions (*dN/dS*) for XX-biased, XY°-biased, and unbiased genes identified at stages G43 and G46. Codes for levels of significance are 0.001 ‘***’, 0.05 ‘*’

We combined data on sex bias from all stages by comparing a set comprised of genes that were XX- or XY°-biased in any stage with the genes that were never sex biased at any stage. In this comparison, the average ratio of non-synonymous to synonymous substitutions (*dN*/*dS*) differed neither between XX- and XY°-biased genes (Wilcoxon test, *W* = 890,990; *p* = 0.40) nor between unbiased and XY°-biased genes (*W* = 1,656,900; *p* = 0.61). The difference was marginally significant between unbiased and XX-biased genes (*W* = 2,692,000; *p* = 0.09). However, stage-specific analyses revealed larger differences, some of them highly significant after correction for multiple testing. At stage G43, unbiased genes had significantly lower *dN/dS* ratios than those biased for either XX females (*W* = 25,589; *p* = 1.3e−05) or XY° males (*W* = 4710; *p* = 0.0002) (Fig. 5). At stage G46, unbiased genes also had significantly lower *dN*/*dS* ratios than those biased for XX females (*W* = 1,320,400, *p* = 0.04; Fig. 5), but not significantly lower than those biased for XY° males (*W* = 823,710, *p* = 0.40). When the analysis was restricted to sex-biased and unbiased genes that were shared between stages G43 and G46, genes biased for either XX females or XY° males showed significantly higher *dN*/*dS* ratios than unbiased genes (Wilcoxon test: XX-biased, *W* = 23,424, *p* = 5.2e−05; XY°-biased, *W* = 3403, *p* = 0.002; Additional file 3: Figure S9a). Interestingly, in the contrast between XY° males and XY° with ovaries at G43, the female-biased genes also showed an elevated rate of evolution (*dN*/*dS*) (Wilcoxon test, *p* = 0.003, Additional file 3: Figure S9b; there were too few male-biased orthologs for meaningful statistics). At G46, furthermore, the XX-male-biased genes showed marginally higher rates of evolution than XX-female-biased genes (Wilcoxon test, *p* = 0.06, Additional file 1: Figure S9c), although neither male-biased nor female-biased genes differed significantly from unbiased genes in terms of *dN*/*dS* ratios (Wilcoxon test, *p* = 0.17, *p* = 0.36 respectively).

**Fig. 6:**
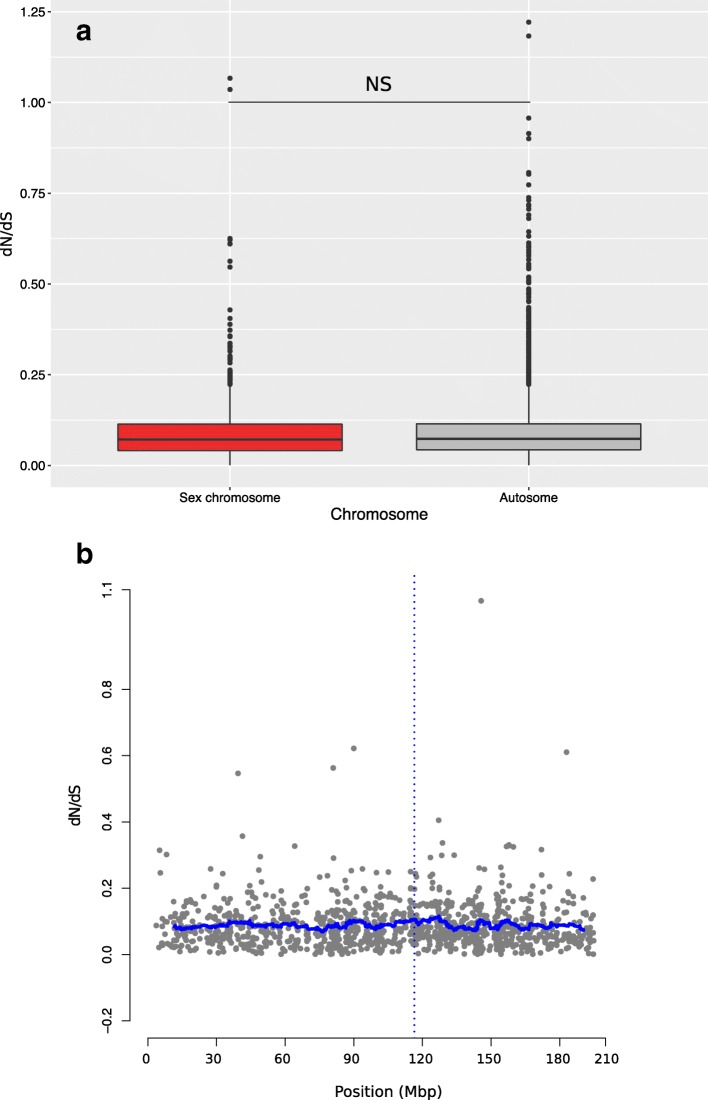
The ratios of non-synonymous to synonymous substitutions *dN/dS* **a** do not differ between sex chromosomes (red) and autosomes and **b** show no special pattern around the sex-determining region (the horizontal blue line shows the average *dN/dS* ratio of a sliding window of 40 genes; *Dmrt1* position marked by the vertical blue dotted line). Codes for significance level is not significant ‘NS’

Genes on the sex chromosome (1110 orthologs) did not differ from autosomal genes (5517 orthologs) in terms of *dN*/*dS* ratio (Wilcoxon test: *W* = 4,191,400; *p* = 0.29, Fig. 6a). Similarly, there was no deviation from the mean *dN/dS* ratio scans with sliding windows of 40 genes along the sex chromosome, including in the region surrounding the candidate sex determining gene *Dmrt1* (Fig. 6b, Additional file 3: Figure S10a, b). Thus, our results provide no evidence for faster-X (or faster sex chromosome) evolution in our system.

## Discussion

Our RNAseq analyses of multiple developmental stages, from one *Rana temporaria* population with proto-sex chromosomes, contributes to our understanding of sex-biased gene expression on three main aspects: (i) the dynamics of sex-biased gene expression across developmental stages, (ii) the signature of selection on sex-biased genes, and (iii) the contribution of proto-sex chromosomes in the buildup of sexual dimorphism throughout development. Below, we discuss these three aspects in turn.

### Dynamics of sex-biased gene expression across developmental stages

The number of sex-biased genes was very low at early stages but drastically increased at metamorphosis, to reach a maximum at the froglet stage whereby 20% of genes were sex-biased in expression (Fig. 1). The near absence of sex bias at G23 is not surprising, since this stage precedes the onset of sex differentiation and gonad development. The very limited sex bias at G27 (9 out of 67,288; 0.01%) and G31 (25 out of 67,288; 0.04%) appears more surprising, given that gonads are thought to display histological differentiation at these stages, according to [40, 41]. However, these studies were conducted in Polish populations that likely belong to the differentiated sex race (N. Rodrigues, pers. comm.), which has early and direct gonadal differentiation. The population studied here belongs to the semi-differentiated sex race in which most XY° juveniles first develop ovaries, replaced by testes by the froglet stage [28, 35, 36], so that genetic sexes might indeed show little differentiation at stage G31. It would be worth expanding our gene expression analyses to populations from the differentiated sex race for comparison. Few studies have addressed sex-biased gene expression in vertebrates at early embryonic stages, prior to the onset of gonad morphological differentiation. In the rainbow trout *Oncorhynchus mykiss* (which also has homomorphic XY sex chromosomes), a larger proportion (8.7%) of genes had sex-biased expression prior to morphological gonad differentiation, though most of them were not related to sexual function [44, 45]. This might indicate an earlier gonadal differentiation in trout but might also stem from differences in methodologies (microarrays versus RNAseq) and sex-bias calling criteria (FDR < 0.2 for the rainbow trout, compared to FDR < 0.05 and |log_2_FC| ≥ 1 in our study).

The drastic increase in the number of sex-biased genes at G43 (1.7%) and G46 (20%) coincides with the morphological differentiation of gonads: two out of seven XY° individuals already had developed testes at G43, and all of them by G46. Our results are consistent with studies of other vertebrates at similar stages of differentiation: in the clawed frog *Xenopus tropicalis*, 1% of genes (588 out of 59,021) were male-biased, and 1.8% (1079 out of 59,021) female-biased by the end of metamorphosis [46]. In chickens, ~ 21% of genes show sex-biased expression (FDR = 0.1) at stages where gonads become morphologically differentiated [4]. Adult stages in vertebrates typically present the strongest sex bias in gene expression (e.g., up to 38% in adult zebra fish [21] and up to 71% in mice [47]). Invertebrates, by contrast, seem to present high degrees of sex bias already at earlier stages: in *Drosophila*, for instance, > 50% of expressed genes at the late larval and pupal stages show moderate to high sex differences [2], similar to the proportions found in adults (50% on average, up to 88% [48, 49]). The earlier expression of sexual dimorphism in invertebrates probably reflects ontogenetic differences with vertebrates, where gonadal ridges first develop as bipotential sex organs, before switching to either testes or ovaries [40, 41]. In contrast, many invertebrates start sexual differentiation soon after fertilization (e.g., *Nasonia* wasps [50]). In holometabolous insects, strong sex bias is expected during metamorphosis occurring at the pupal stage, when the body is entirely restructured into male or female adults [18].

We also detected very little overlap of sex-biased genes between stages suggesting a rapid turnover during development, a situation similar to that found in chickens [4] and rainbow trout [45]. This contrasts again with *Drosophila* where most sex-biased genes are consistent across larval and pupal stages [2]. It is tempting to also interpret this contrast in the context of differences in the patterns of sexual differentiation between vertebrates and invertebrates (though this remains largely speculative, given the limited number of studies available for comparison).

The few sex-biased genes at the pre-metamorph stages showed a trend towards XY°-biased expression (8 out of 9 at G27, 15 out of 25 at G31), which differs from the rainbow trout data, where equal numbers of male- and female-biased genes were reported during early embryonic stages [45]. At later stages (G43 and G46), however, sex bias was strongly and significantly skewed towards XX females, both in terms of gene numbers and expression ratios. This is in line with data from *X*. *tropicalis*, which showed consistent female bias in gene expression during metamorphosis [46], as well as from chickens, with dominantly female-biased gene expression during the morphological differentiation of gonads [4]. The same occurs in the *Drosophila* larval and pre-pupal stages, during which gonads already show morphological differentiation [2]. Taken together, these studies suggest that female bias in gene expression seems dominant during the morphological differentiation of gonads, although the directions of bias prior to this morphological differentiation may vary across species. This consistency across studies and taxa in the amount, direction, and timing of sex bias also suggests that our use of whole body (rather than gonads) for RNAseq analyses, associated with stringent criteria when calling sex-biased genes, had no major effect on conclusions. The drastic increase in sex bias at stages G43 to G46 is likely to reflect the patterns of gonadal development, as many GO terms of sex-biased genes at these two stages are related to reproduction. Other GO terms included sex steroids at stage G46 (Additional file 5), which have also been detected in the brain tissue or whole body of other frog species at pre-metamorphosis, during metamorphosis, and towards adulthood (e.g., *Rana pipiens*, *Xenopus tropicalis*, and *Physalaemus pustulosus* [51–53]).

Our study population belongs to the semi-differentiated sex race, in which some XY° individuals first develop ovaries, which are replaced by testes by the froglet stage, to result in adult phenotypic males. At G43, the two XY° males had patterns of gene expression well differentiated from the four XX females. In contrast, the five XY° individuals still with ovaries at G43 did not constitute a homogeneous gene expression group. Two of them were more similar to the XY° males: they had the same low expression levels at gene set 1 (enriched in genes with reproductive and immune functions, Fig. 4b), but were otherwise similar to females. The three remaining individuals clustered with XX females, displaying an overall female-like expression profile, except for gene set 3 where they had the same low expression profile as males. This suggests either different steps in the process of transition towards the male phenotype or possibly different pathways towards fully differentiated male phenotypes. It would also be worth comparing these patterns with data from the differentiated sex race, where we predict an earlier, more homogeneous, and better-canalized transition to maleness.

### Signatures of selection on sex-biased genes

A higher between-species sequence divergence at sex-biased genes is thought to reflect sex-specific evolutionary pressures acting on the loci underlying sexually dimorphic traits (reviewed in [3]). We found little differences in *dN/dS* ratio between genes that were sex biased at some stage and genes that were not sex biased at any stage. This likely results from the rapid turnover in sex bias of most genes, since sex-biased genes consistently show elevated *dN/dS* ratio compared to unbiased genes in stage-specific comparisons (Fig. 5). This consistent signature of selection across stages towards increased evolutionary rate for sex-biased genes differs from the situation found in chickens, where patterns of divergence of sex-biased genes varied across stages [4]. Interestingly, when calling sex bias between XY° males and XY° individuals with ovaries at G43, we found that genes biased for XY° “females” had a higher rate of evolution than unbiased genes (Additional file 3: Figure S9b). Furthermore, using one XX male to call sex bias at G46, we found that XX female-biased genes evolved marginally slower than XX male-biased genes at G46 (Additional file 3: Figure S9c). These results suggest that sexual selection acts on sex-biased genes based on phenotypic rather than genotypic sex. In support, we found the vast majority of sex-biased genes are shared when contrasting XX females or XY° females with XY° males (202 out of 203; Additional file 3: Figure S11), and a majority of female-biased (6433 out of 6473) and male-biased (2283 out of 2285) genes when contrasting XX females with either the XX or XY° males at G46 (Additional file 3: Figure S7a, b).

We also found fewer *X. tropicalis* orthologs for male-biased genes than for female-biased or unbiased genes (especially at G46 which had the highest number of male-biased genes). One reason for this might be that a higher proportion of male-biased genes may be too diverged from *X. tropicalis* to generate a significant BLAST hit, meaning they will be underrepresented in the ortholog set. This would make their calculated *dN/dS* an underestimation. Faster male evolution is expected both from stronger sexual selection [4, 54, 55] and from relaxed purifying selection on males (faster-male effect, reviewed in [3]). Overall, our study unveils clear signatures of sex-specific evolutionary pressures acting on dimorphic traits, at developmental stages in which gonads show morphological differentiation. This implies that the genes identified here as sex biased have been involved in sexual dimorphism over evolutionary times long enough for sex-selective pressures to translate into higher *dN/dS* ratios.

### Sexualization of proto-sex chromosomes

Unlike studies of organisms with differentiated sex chromosomes (reviewed by [3]), we found no clear evidence for sexualization of the proto-sex chromosomes in *R. temporaria*, even at developmental stages that display strong sex bias in gene expression. (i) Sex-biased genes were not more common on proto-sex chromosome than on autosomes, nor around the candidate sex-determining locus compared to the rest of the sex chromosome. Male-biased genes at G46 constituted a lower proportion of sex-biased genes on sex chromosomes than on autosomes, which might indicate early feminization. However, the effect was weak and might also result from faster evolution of male-biased genes (and hence lower detectability). (ii) There was no difference between sex chromosomes and autosomes in the ratio of male-to-female expression over all identified orthologs. Similarly, there was no difference when analyzing separately the genes with significant male- or female-biased expression, and their distribution was uniform along the sex chromosome. (iii) We found no evidence for faster-X effect, as the *dN/dS* ratio did not differ between sex-linked and autosomal genes, and no specific pattern was found along the sex chromosomes. (iv) We found negligible differences in expression between XX and XY° males at G46: only 0.06% of genes had significantly different expression (with two orthologs found on autosomes). The |log_2_| ratio of XY° to XX expression also did not differ between autosomal and sex-linked orthologs and had uniform distribution along the sex chromosome, suggesting negligible X-Y° differentiation. This is consistent with the results from expression patterns showing that the XX with testes clusters with XY° males, well apart from XX females. Not only does this confirm the absence of degeneration along the proto-Y chromosome, but it also shows that sex differences in expression only depend on phenotypic sex, not on genotypic sex.

The absence of faster-X effect in our study is consistent with the absence of faster-Z effect in the nascent sex chromosomes of the basket willow *Salix viminalis*, which likely represents a more advanced stage of sex-chromosome differentiation than the common frog as it shows evidence for Z-W differentiation and masculinization of Z-expression in the sex-determining region [27]. More studies on organisms with young sex chromosomes at different stages in their evolution are needed to fully specify the sequential steps of differentiation that accompany the birth of sex chromosomes.

## Conclusions

Our data suggest no role for the proto-sex chromosomes of *Rana temporaria* in the buildup of sexual dimorphism, which is likely to result instead from the differential expression of autosomal genes. This conclusion is in line with the evidence of fully functional XX males and XY females in natural populations of common frogs [33, 34]. Autosomal control of sex dimorphism certainly facilitates the dynamics of sex chromosomes, which display both within-species polymorphism and high turnover rate in Ranidae (e.g., [56–58]): sexual dimorphism depending on sex-linked genes would strongly oppose such transitions in sex chromosome [59, 60]. More generally, our results challenge the common idea that sexually antagonistic genes accumulate on nascent sex chromosomes and play a central role in their ensuing evolution (e.g., [6, 61, 62]).

## Methods

### Field sampling and rearing conditions

Six mating pairs in amplexus, as well as 18 females and 22 males, were caught during the 2015 breeding season in the southern Swedish breeding pond of Stensma (55°50′51.83″ N, 13°55′24.83″ E), 48 km northeast of the previously studied population of Tvedöra [28, 31]. The single adults were sampled for buccal cells with sterile cotton swabs and immediately released at the place of capture. The six mating pairs were left overnight in 11-l plastic tanks to lay their clutch. On the next day, they were similarly sampled for buccal cells and released at the place of capture. The six clutches were brought back to the University of Lausanne, and the six families were reared in separate tanks in a climatic room at constant conditions (19 °C with 12:12 light to dark cycle), in order to minimize environmental effects on gene expression. Juveniles were first fed fish flakes, then fruit flies, and small crickets after metamorphosis. Two to four offspring from each clutch were sampled at each of five developmental stages [37], namely stages G23, G27, G31, G43 (metamorph; 1.2–1.4 cm snout-vent length), and G46 (froglet; 2.1–2.3 cm snout-vent length), which at our rearing conditions took place respectively 10 days, 12 days, 27 days, 3 months, and 6 months after spawning. These stages represent important points regarding sex determination and differentiation [40, 41]: gonadal development is first initiated at stage G27, with histological differentiation visible from stage G31, and morphological differentiation from stage G43 (metamorphosis). At stage G46, the secondary differentiation of males should be mostly achieved, with ovaries entirely replaced by testes ([35]; see Introduction). Sampled juveniles were anesthetized and euthanized in 0.2% ethyl3-aminobenzoate methanesulfonate salt solution (MS222), then immediately plunged in RNAlater (Qiagen). The tail tip from each tadpole and a toe clip from metamorphs and froglets were cut for genotyping. Samples of the two latter stages (G43 and G46) were dissected for phenotypic sex determination (see below), and their digestive tracts (stomach, small intestine, large intestine) were removed to limit contamination of RNA analyses by food remains and microorganisms. Samples in RNAlater were preserved at − 20 °C up to 10 months before RNA extraction.

### Genotyping

The genotypic sex of single adults and parents was determined based both on four *Dmrt* markers with Y-diagnostic alleles (namely *Dmrt1–1*, *Dmrt1–2*, *Dmrt1–5*, and *Dmrt3*) and on 14 sex-linked anonymous microsatellites (*Bfg147*, *Rtemp5*, *RtSB03*, *Bfg021*, *Bfg266*, *RtuB*, *Bfg093*, *Bfg191*, *Bfg053*, *Bfg172*, *Bfg131*, *Bfg092*, *Bfg072*, *Kank1*) with alleles diagnostic of fully differentiated Y chromosomes (primer sequences from [28, 31]; Additional file 1). As none of the parents had a fully differentiated Y chromosome, progenies were only genotyped with the *Dmrt* markers. After an overnight treatment at 56 °C with tissue lysis buffer ATL and 20% proteinase K (Qiagen), PCR reactions were performed in a total volume of 10 μl, including 3 μl of extracted DNA, 2.22 μl of Milli-Q water, 3 μl of Qiagen Multiplex Master Mix, and 0.14 to 0.3 μl of labeled forward primer and 0.14 to 0.3 μl of unlabeled reverse primer (in total 1.78 μl of primer mix). PCRs were conducted on Perkin Elmer 2700 machines using the following thermal profile: 15 min of Hot Start Taq polymerase activation at 95 °C, followed by 35 cycles including denaturation at 94 °C for 30 s, annealing at 55 °C for 1.5 min, and elongation at 72 °C for 1 min, ending the PCR with a final elongation of 30 min at 60 °C. PCR products were then analyzed on an automated ABI Prism 3100 sequencer (Applied Biosystems, Foster City, CA, USA), and alleles were scored using GeneMapper v. 4.0 (Applied Biosystems).

### Phenotypic sex

The phenotypic sex of G43 and G46 samples was determined based on gonad morphology, following dissection in RNAlater (Qiagen) under a binocular microscope. Ovaries in common frogs develop from the whole gonadal primordia into a large whitish/yellowish structure with distinct lobes and a characteristic granular aspect conferred by the many oocytes embedded in the cortex [40]. In contrast, testes develop from the anterior part of the gonadal primordia only (the posterior part degenerates) into a small oblong structure, with a smooth cortex covered with melanic spots [41]. Each individual was scored as phenotypic male, female, or undifferentiated, following the gonad-scoring description in (Additional file 1) [31].

### RNA extraction and sequencing

In order to maximize independence of biological replicates, we selected for each stage at least one XX and one XY° individual from each of three to six clutches, based on the genotyping results (Additional file 2), resulting in a total of 46 RNA samples across five developmental stages. RNA was extracted from whole bodies for the earliest three stages, because individuals are too small to reliably extract RNA from particular tissues. For the later stages G43 and G46, whole bodies were also used in order to have comparable datasets with the earliest three stages. RNAseq analyses are thus expected to capture allometric differences of organs between stages. RNA extractions were performed following a mixed Trizol/Qiagen columns protocol. We followed the normal Trizol protocol until the two-phase stage (apolar and aqueous phase). We took 500 μl of the aqueous phase, added 300 μl of ethanol, and loaded the mix in an RNeasy column (Qiagen), then followed the standard Qiagen RNeasy protocol. Each RNA-later preserved sample was individually homogenized in Trizol (Life Technologies), followed by phase separation (using chloroform). After ethanol precipitation of the upper phase, RNA was washed with 70% ethanol twice and collected, followed by a DNase digestion step. RNA libraries were then prepared and barcoded at the Lausanne Genomic Technologies Facility, University of Lausanne, using standard protocols. Six RNA libraries were multiplexed per lane and were sequenced on an Illumina HiSeq 2500 resulting in, on average, 84.2 million 100-bp paired-end reads per sample.

### De novo transcriptome assembly, mapping, and annotation

RNAseq reads were quality assessed using FastQC v0.11.2 (https://www.bioinformatics.babraham.ac.uk/projects/fastqc/) and quality trimmed using Trimmomatic v0.33 with default parameters for paired-end reads [63]. We filtered reads containing adaptor sequences and trimmed reads if the sliding window average Phred score over four bases was < 15 or if the leading/trailing bases had a Phred score < 3. Reads were then removed post filtering if either read pair was < 36 bases. In order to include all possible combinations of stage, phenotypic sex, and genotypic sex in the de novo transcriptome assembly, we used one XY° and one XX individual per stage, except for stage G43 where two individuals each of XX females, XY° males, and XY° with ovaries were sampled (i.e., 14 samples in total), using Trinity v2.4.0 with default parameters [64]. De novo transcriptome assemblies typically consist of more contigs than can possibly be considered “real,” even when alternative splicing is taken into account [14]. We thus applied a series of filtering steps to reduce the number of erroneous and non-expressed contigs. First, we removed transcripts shorter than 300 bp. We then mapped all the reads from all 46 samples to the most expressed Trinity isoform per gene cluster, using Kallisto v0.43.0 [65]. We applied a minimum expression filter of 1 for trimmed mean of the log expression ratios (trimmed mean of *M* values, TMM; mapping results from Kallisto output). Haplotype merging was then applied based on 90% transcript identity using cd-hit v4.6.1 (cd-hit-est for DNAs clustering, http://weizhongli-lab.org/cd-hit/). Finally, we removed mapped ERCC internal control and ribosomal rRNA transcripts. After filtering, 67,288 transcripts remained. We used BUSCO v2 [38] with the tetrapoda database to assess the completeness of the filtered transcriptome and Bowtie2 [39] to evaluate the percentage of the total reads which could be mapped to the assembled transcriptome. The transcriptome and gene ontology were annotated using Trinotate v3.0.2 (https://trinotate.github.io), using default parameters.

### Sex-biased gene expression analysis

To quantify gene expression, we mapped the trimmed reads of all 46 samples to the filtered assembled transcriptome with Kallisto v.0.43.0 [65]. Read counts of the output from Kallisto mapping were imported for gene expression analysis in EdgeR v3.4 [66, 67]. We filtered the low counts and kept genes with average Log_e_(CPM) > 0 per sample and CPM > 1 in at least half of the samples for each genetic sex per developmental stage. We then normalized the expression by trimmed mean of *M* values (TMM). We explored the libraries per stage in two dimensions using multi-dimensional scaling (MDS) plots (Additional file 3: Figure S12a, b, c, d, Figure S6). Normalized expression counts for each sample were used to calculate sex bias using standard measures. We first identified sex-biased genes based on overall expression of each comparison group and using Benjamini-Hochberg correction for multiple testing with false discovery rate (FDR) of 5%. We identified sex-biased genes for each developmental stage separately. Sex bias was classified into four categories of fold changes, namely 2 (low), 2–4 (mild), 4–8 (high), and > 8 (very high), and expressed as log_2_ ratio of male-to-female expression (which has negative values for female-biased genes and positive values for male-biased genes). As suggested by [68], only fold changes ≥ 2 will be interpreted throughout, in order to minimize possible scaling issues due to whole-body sampling (ovaries are slightly larger than testes, which may potentially lead to bias in calling sex-biased gene expression). Thus, unless stated otherwise, both conditions FDR < 0.05 and |log_2_FC| ≥ 1 will have to be met when calling sex bias. The sex-biased genes at stages G43 and G46 were defined after excluding the sex-reversed individuals (only XX females and XY° males were used) to eliminate possible noise induced by sex reversals, unless otherwise stated.

### Hierarchical clustering and heatmaps

Hierarchical clustering was performed using distance matrix (Euclidean clustering method) with the R package dynamicTreeCut [69], using complete linkage in the R package pvclust [70], with bootstrap resampling (10,000 replicates). Differentially expressed genes were identified based on log_2_ of XY°-male-to-XX-female expression (with a FDR threshold of 0.05). Heatmaps were generated separately for G43 and G46, and expression values (logCPM) for each differentially expressed gene (per row) were plotted using the heatmap.2 function in the R package gplots (R v3.4.0).

### Gene ontology

To determine whether particular classes of genes were enriched for certain functional characteristics, we conducted a Gene Ontology (GO) enrichment analysis separately for genes showing differential expression between categories of individuals. Gene ontology annotation was obtained from Trinotate (https://trinotate.github.io). GO term enrichment analysis was conducted with TopGO [71]. Enrichment was determined at the 0.05 threshold for *p* values resulting from Fisher’s exact tests that account for GO term topology (with topGO algorithm “weight01”).

### Sequence divergence of sex-biased and sex-linked genes

Candidate coding regions within transcript sequences were identified from the transcriptome using TransDecoder v2.0.1 (https://github.com/TransDecoder/TransDecoder). If multiple open reading frames (ORFs) were detected for a transcript, we used the longest one. This resulted in 28,222 ORFs in total. Coding DNS sequence (CDS) of *Xenopus tropicalis* were downloaded from XenBase (http://www.xenbase.org/other/static/ftpDatafiles.jsp). Given the strong chromosome-level gene synteny between *R. temporaria* and *X. tropicalis* [42, 43], we performed a reciprocal best BLAST of coding sequences between the two species (custom perl script, protein sequence comparison with an e-value cutoff of 1e−10 and minimum percentage identity of 30% [26]) to identify orthologs and assign the location of each transcript on the genome. In total, 10,756 reciprocal 1:1 orthologs were identified across the genome.

Reciprocal orthologs were aligned with PRANK (v140603) using the codon model [72]. Each alignment was then analyzed with codeml in PAML [73] (runmode − 2) to calculate the number of nonsynonymous substitutions per nonsynonymous site (*dN*), the number of synonymous substitutions per synonymous site (*dS*), and the ratio of the two (*dN*/*dS*). As mutational saturation and double hits can lead to inaccurate divergence estimates [74], orthologs were excluded if *dS* > 2. We then compared *dN*/*dS* ratio among female-biased, male-biased, and unbiased genes at each developmental stage. To assess the differences of *dN*/*dS* ratios between sex-biased and unbiased genes, as well as between female-biased and male-biased genes, datasets were compared using a non-parametric Wilcoxon test for each developmental stage when applicable. To compare the differences between each two groups, multiple comparisons among groups were done using the Tukey test as implemented in the R function package for general linear hypothesis [75]. Similarly, to assess possible faster-X effects, we compared *dN/dS* ratio of orthologs from sex chromosome and autosomes. We compared the differences between the two groups with a Wilcoxon test. All statistics were performed in R v3.4.0 [76].

### Assessing transcriptional degeneration of proto-Y chromosome

To investigate possible transcriptional degeneration of the proto-Y chromosome, we used a Wilcoxon test to compare the expression of all genes on sex chromosome and autosomes between XY° and XX males at stage G46 (log_2_ (XY°/XX)).

### Sliding window analysis

Moving averages of gene expression ratios/sequence divergence were calculated in R v3.4.0 [76], based on sliding window analysis using the Rollapply function in the Zoo R package. Window size was 40 genes at G46 [77] but 20 genes at G43 due to the lower number of sex-biased genes (Additional file 3: Figure S5a, b).

## Additional files

Additional file 1: All LG2 marker and *Dmrt1* genotype of adults and progeny samples used in RNAseq analysis. (XLSX 32 kb)

Additional file 2: Results of SuperExactTest for shared XY^0^-biased genes. (XLS 41 kb)

Additional file 3: All supplementary figures. (PDF 3566 kb)

Additional file 4: **Table S1.** Genomic location distributions of one-to-one ortholog of reciprocal best BLAST hit between *R. temporaria* and *X. tropicalis*. *Ratio between *R. temporaria* ortholog number and the number of *X. tropicalis* gene number per chromosome. **Table S2.** GO enrichment analysis for differentially expressed gene clustering groups at stage G43. GO depicts three complementary biological concepts including biological process (BP), molecular function (MF), and cellular component (CC). (DOC 98 kb)

Additional file 5: GO term and Fisher’s exact test for significance at each developmental stage. (XLSX 60 kb)

## Abbreviations

G23, G27, G31, G43, G46: Gosner stages 23, 27, 31, 43, and 46
FDR: False discovery rate
GO: Gene ontology
*Dmrt1*: Doublesex and mab-3 related transcription factor 1 gene
BLAST: Basic local alignment search tool
CDS: Coding DNA sequences
TMM: Trimmed mean of *M* values
CPM: Count per million
