# Supplementary material for: Evolutionary and developmental dynamics of sex-biased gene expression in common frogs with proto-Y chromosomes
Source: Genome Biol. 2018 Oct 5;19:156. doi: 10.1186/s13059-018-1548-4 (PMC6173898; doi:10.1186/s13059-018-1548-4)
Supplement: Supplementary file 3 — All supplementary figures. (PDF 3566 kb) [file 13059_2018_1548_MOESM3_ESM.pdf]

Evolutionary and developmental dynamics of sex-biased gene expression in common frogs with proto-Y chromosomes

Wen-Juan Ma, Paris Veltsos, Roberto Sermier, Darren J. Parker, Nicolas Perrin

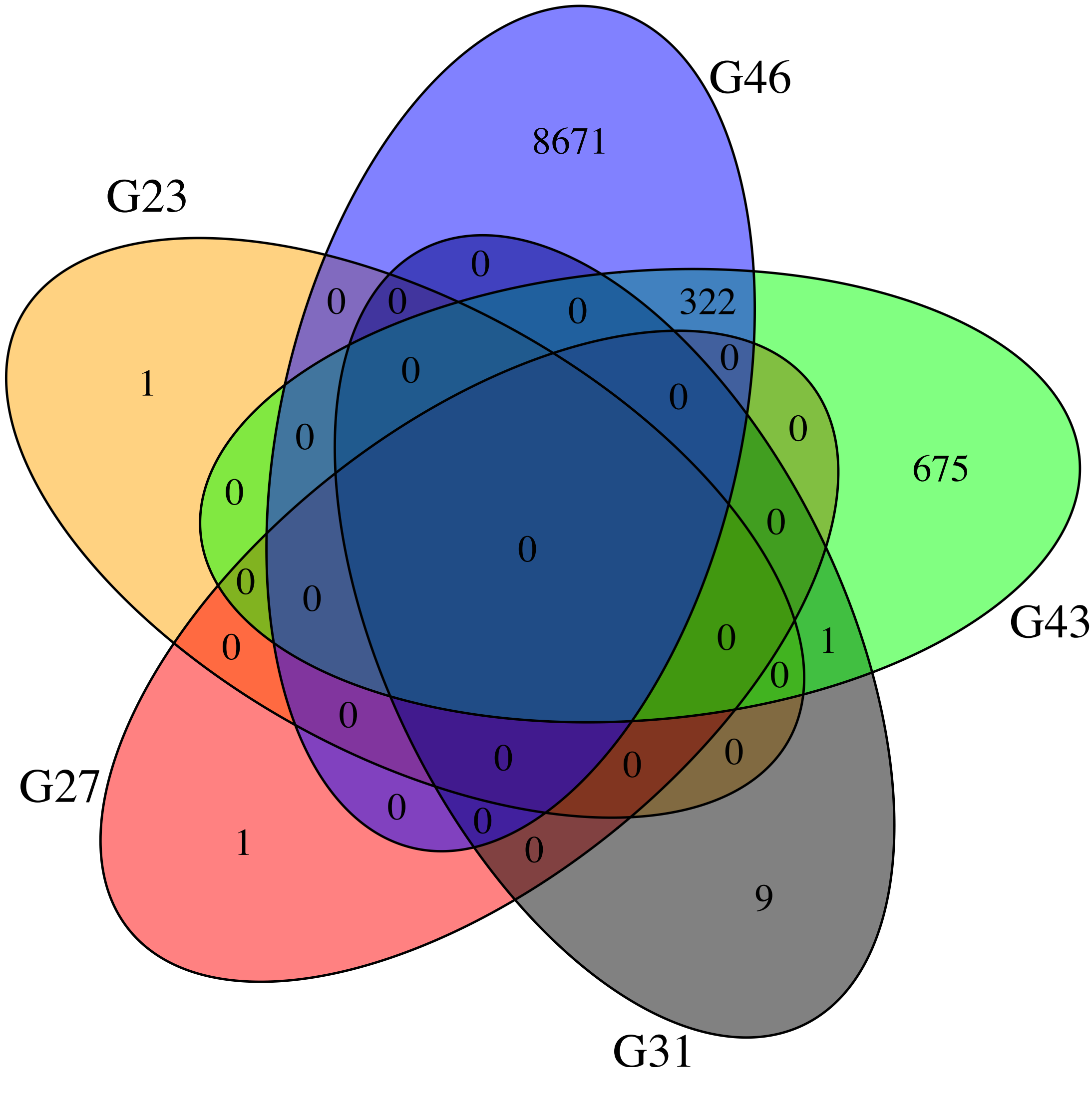

(a)

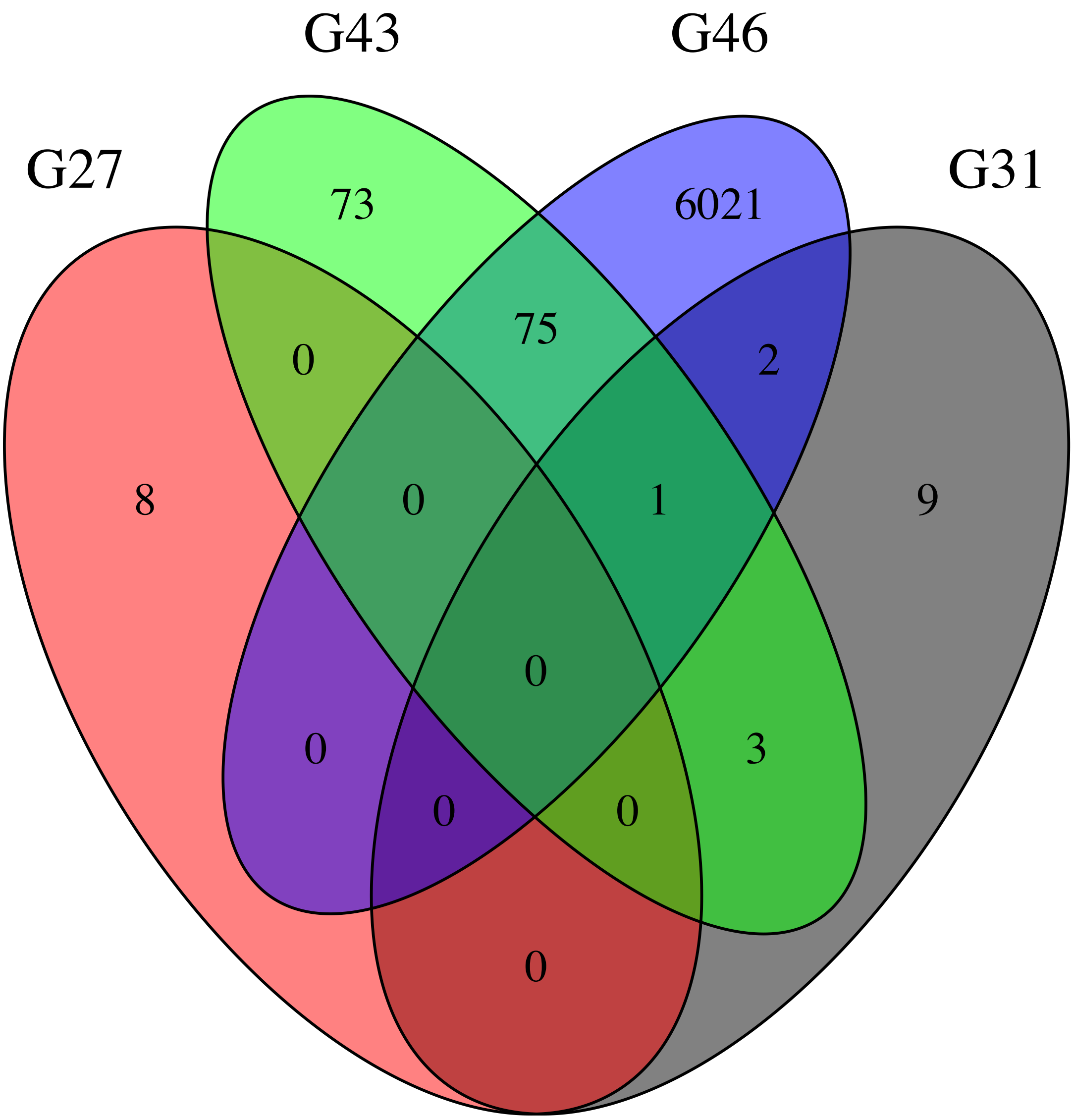

(b)

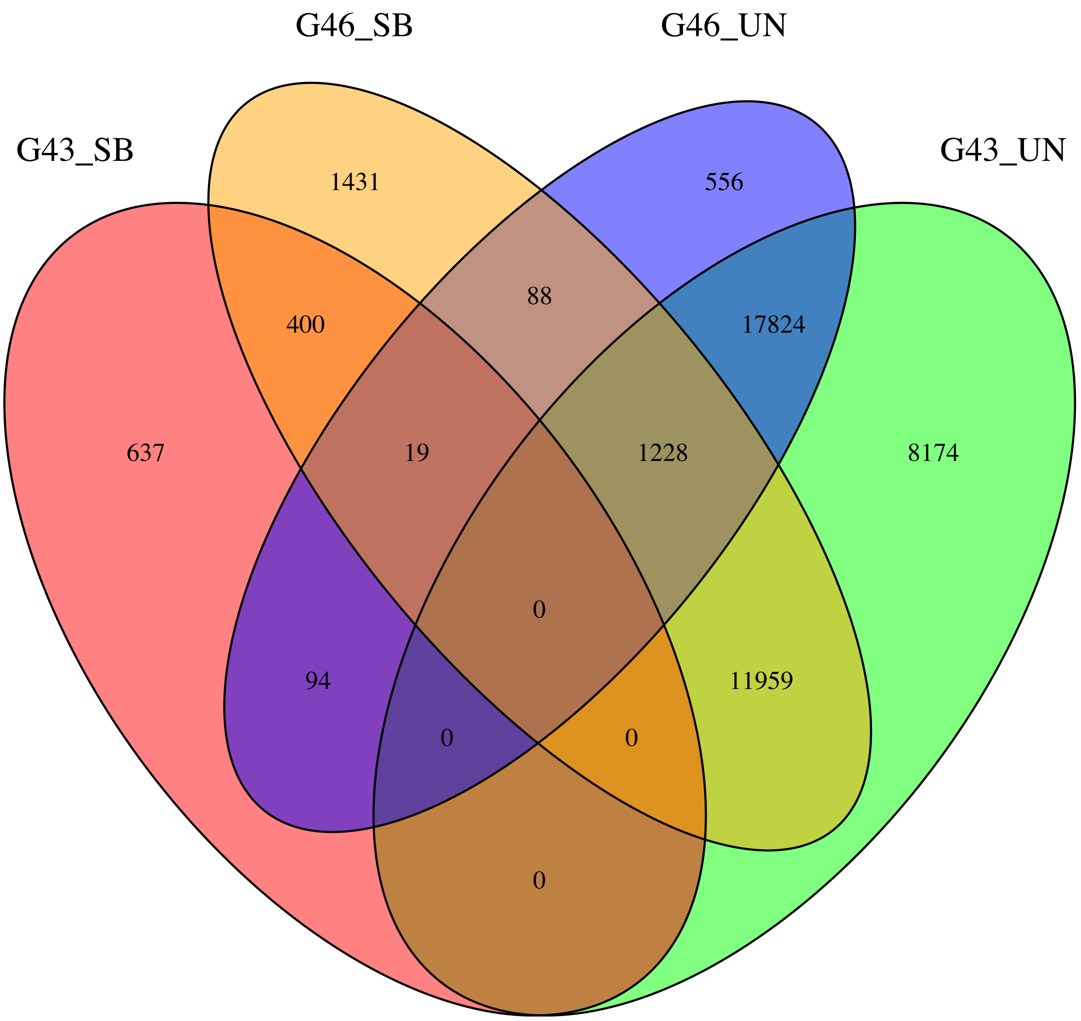

(c)

Figure S1. Venn diagram of XX-biased genes (a), XY^0-biased genes (b) across five developmental stages, and sex-biased (G43_SB, G46_SB) and unbiased genes (G43_UN, G46_UN) between the two stages G43 and G46 (c). We use XX females and XY^0 males to identify sex bias at stage G43 and G46. Sex-biased genes with fold change ≥2, FDR < 0.05 are reported. No XY^0-biased genes were detected at stage G23 (as shown in b).

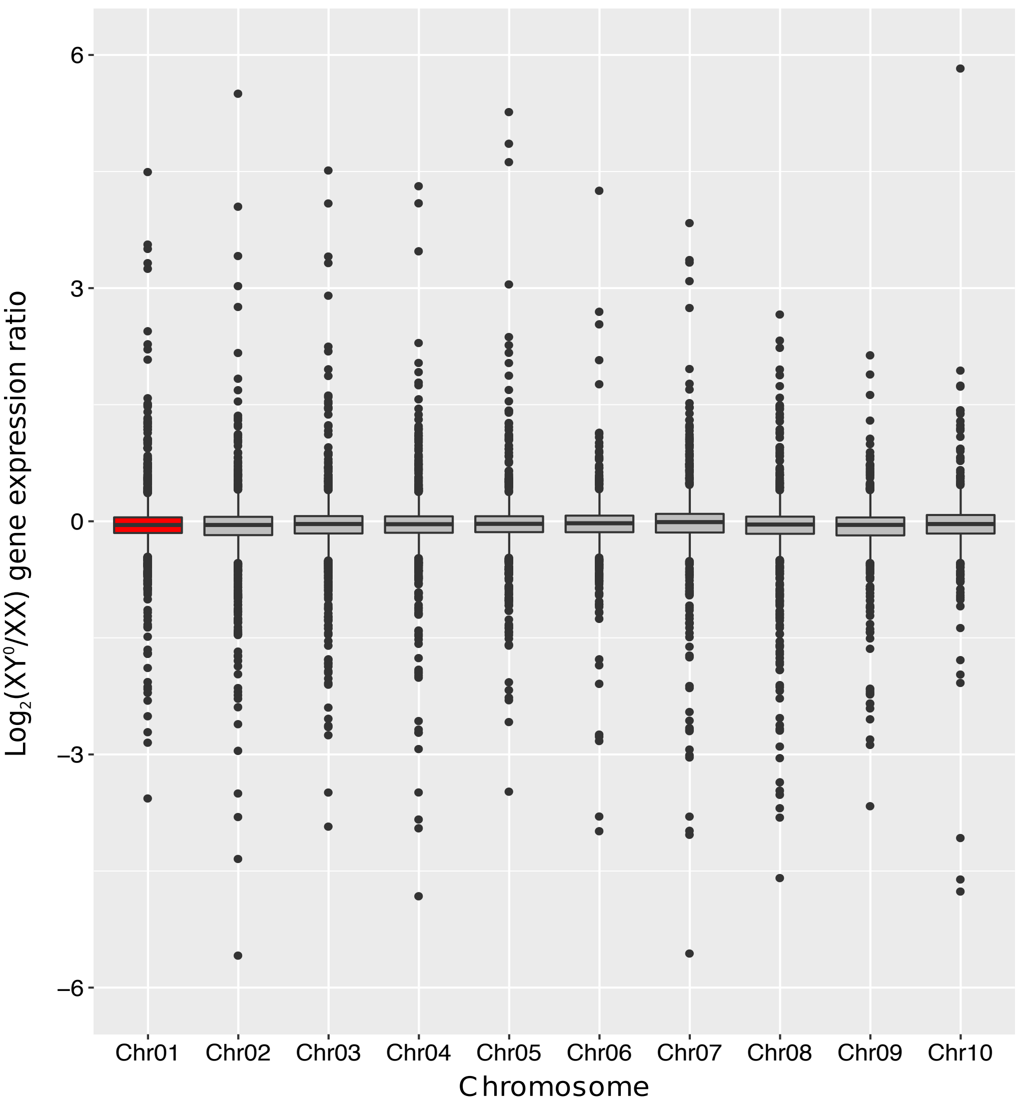
6
3
0
−3
−6
Log2(XY0/XX) gene expression ratio
Chr01
Chr02
Chr03
Chr04
Chr05
Chr06
Chr07
Chr08
Chr09
Chr10
Chromosome

(a)

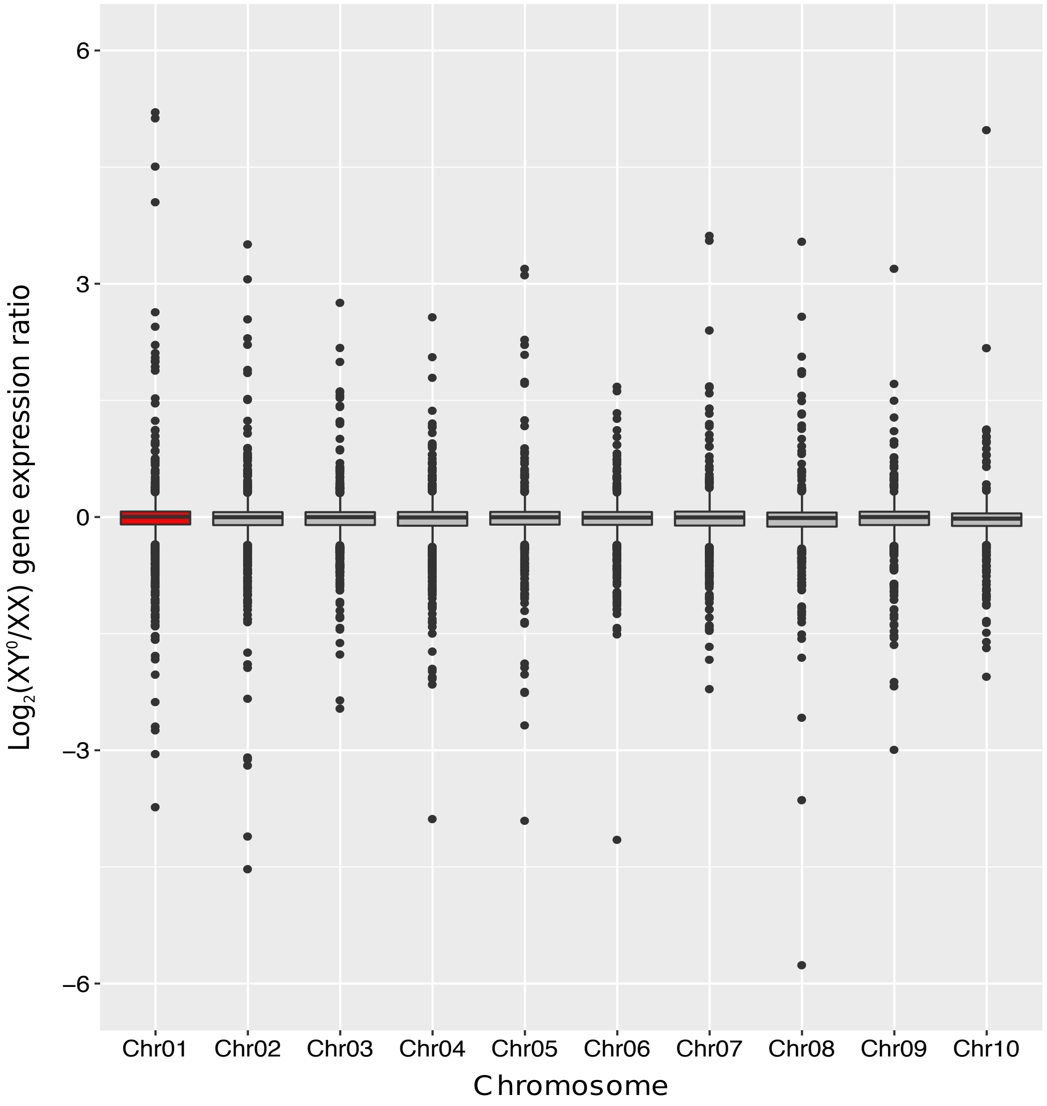

(b)

Figure S2. $Log_2(XY^0/XX)$ gene expression ratio over all orthologs (on *X. tropicalis*) across the genome at both stages G43 (a) and G46 (b). Sex chromosome is Chr01 (red, on the left), and autosomes are Chr02 to Chr10 (grey).

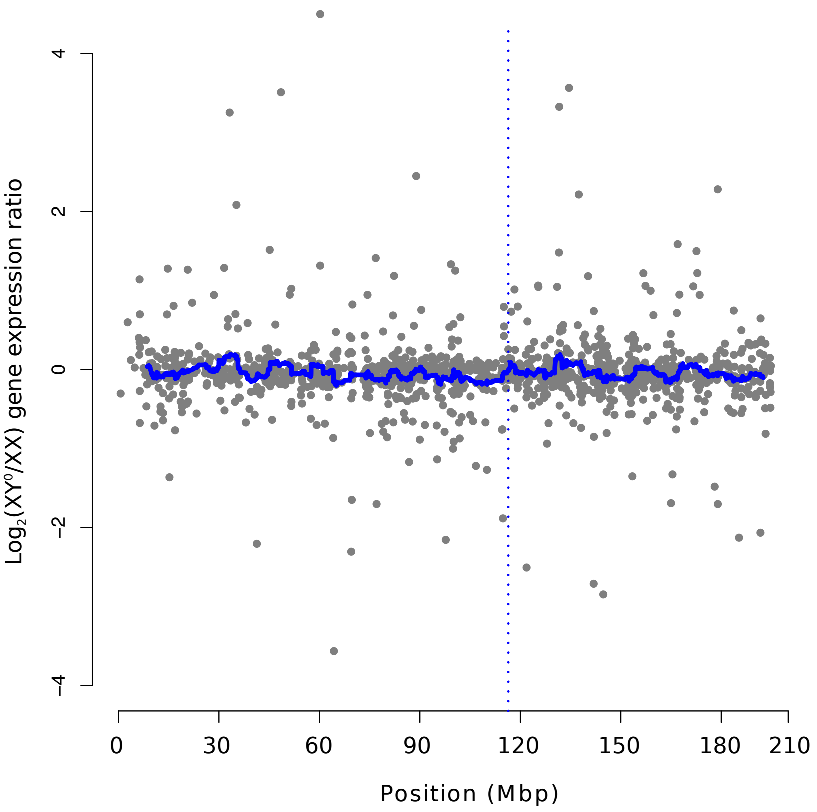
Log2(XY0/XX) gene expression ratio
4
2
0
−2
−4
0
30
60
90
120
150
180
210
Position (Mbp)

(a)

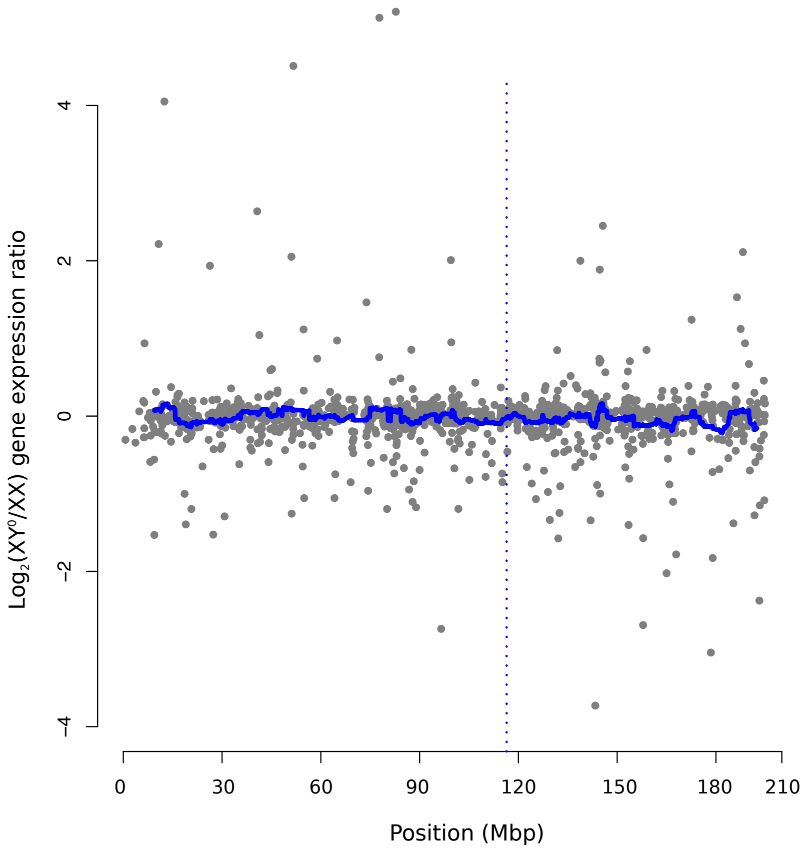
Log2(XY0/XX) gene expression ratio
4
2
0
−2
−4
0
30
60
90
120
150
180
210
Position (Mbp)

(b)

Figure S3. $Log_2(XY^0/XX)$ gene expression ratio over orthologs (on *X. tropicalis*) along the sex chromosome at stage G43 (a) and G46 (b). Both show no special pattern around the sex-determining region. The horizontal blue line shows the average gene expression ratio of a sliding window of 40 genes, and *Dmrt1* position is marked by the vertical blue dotted line.

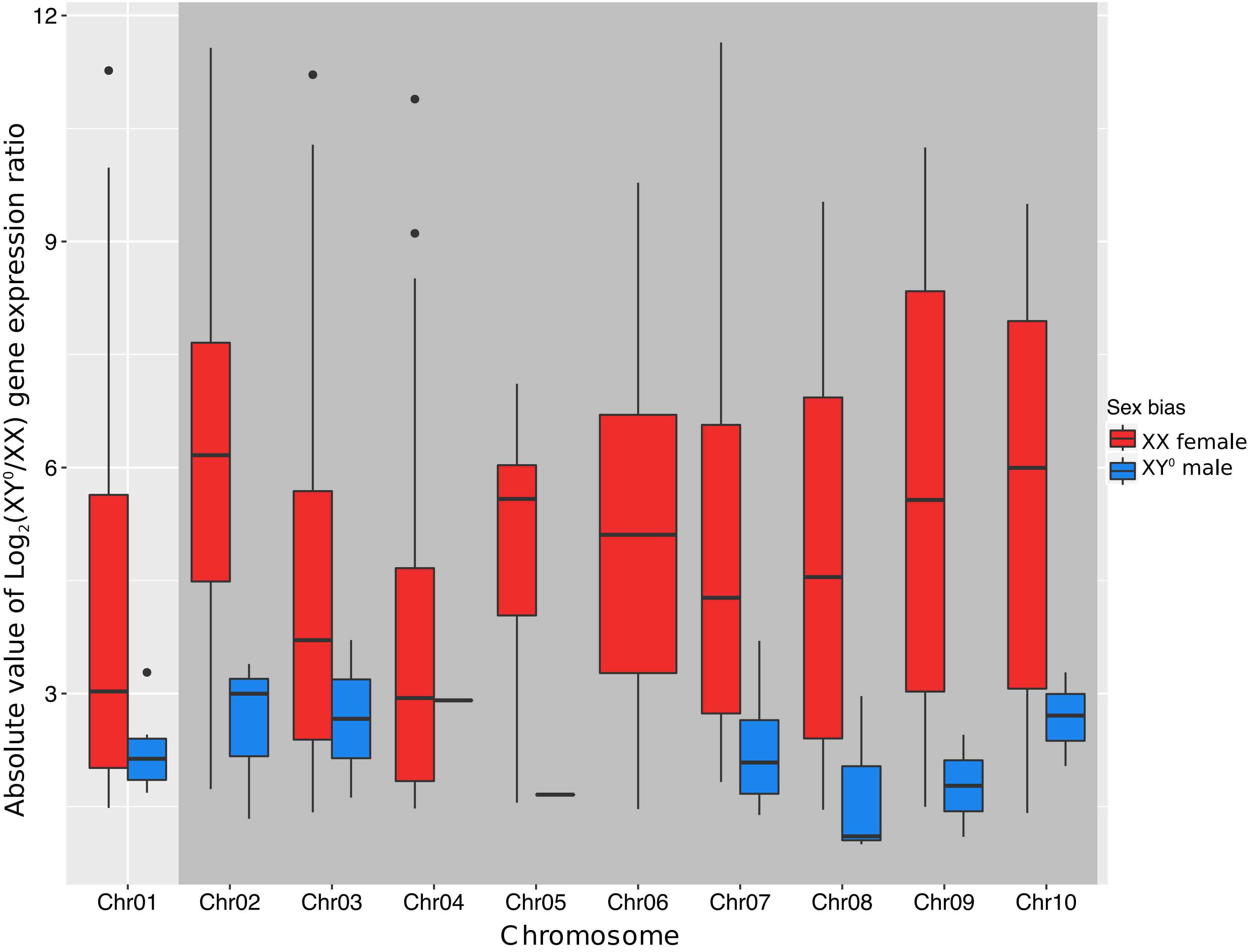

12
9
6
3
Absolute value of Log2(XY0/XX) gene expression ratio
Chr01
Chr02
Chr03
Chr04
Chr05
Chr06
Chr07
Chr08
Chr09
Chr10
Chromosome
Sex bias
XX female
XY0 male

(a)

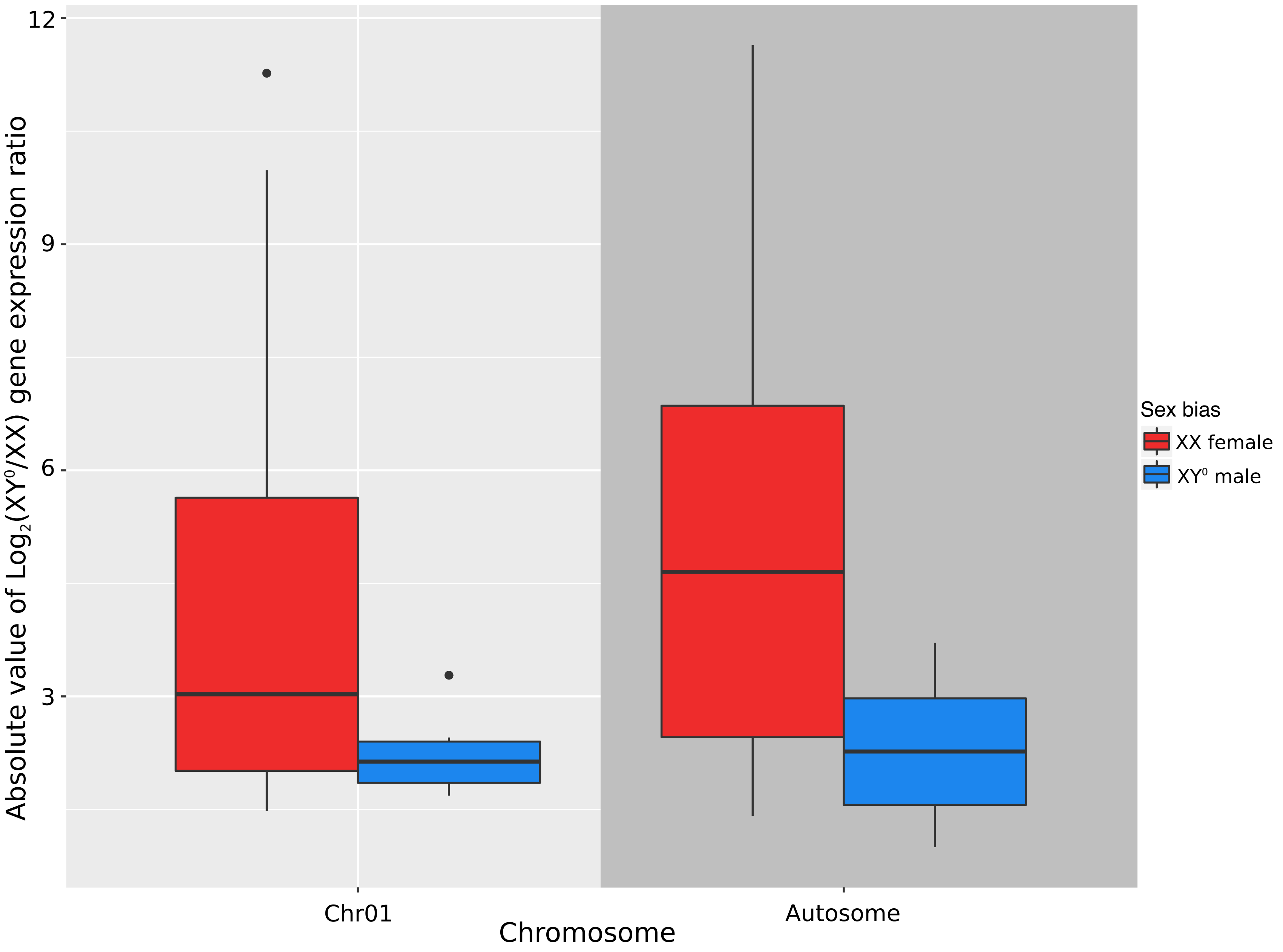
12
9
6
3
Absolute value of Log2(XY0/XX) gene expression ratio
Chr01
Autosome
Chromosome
Sex bias
XX female
XY0 male

(b)

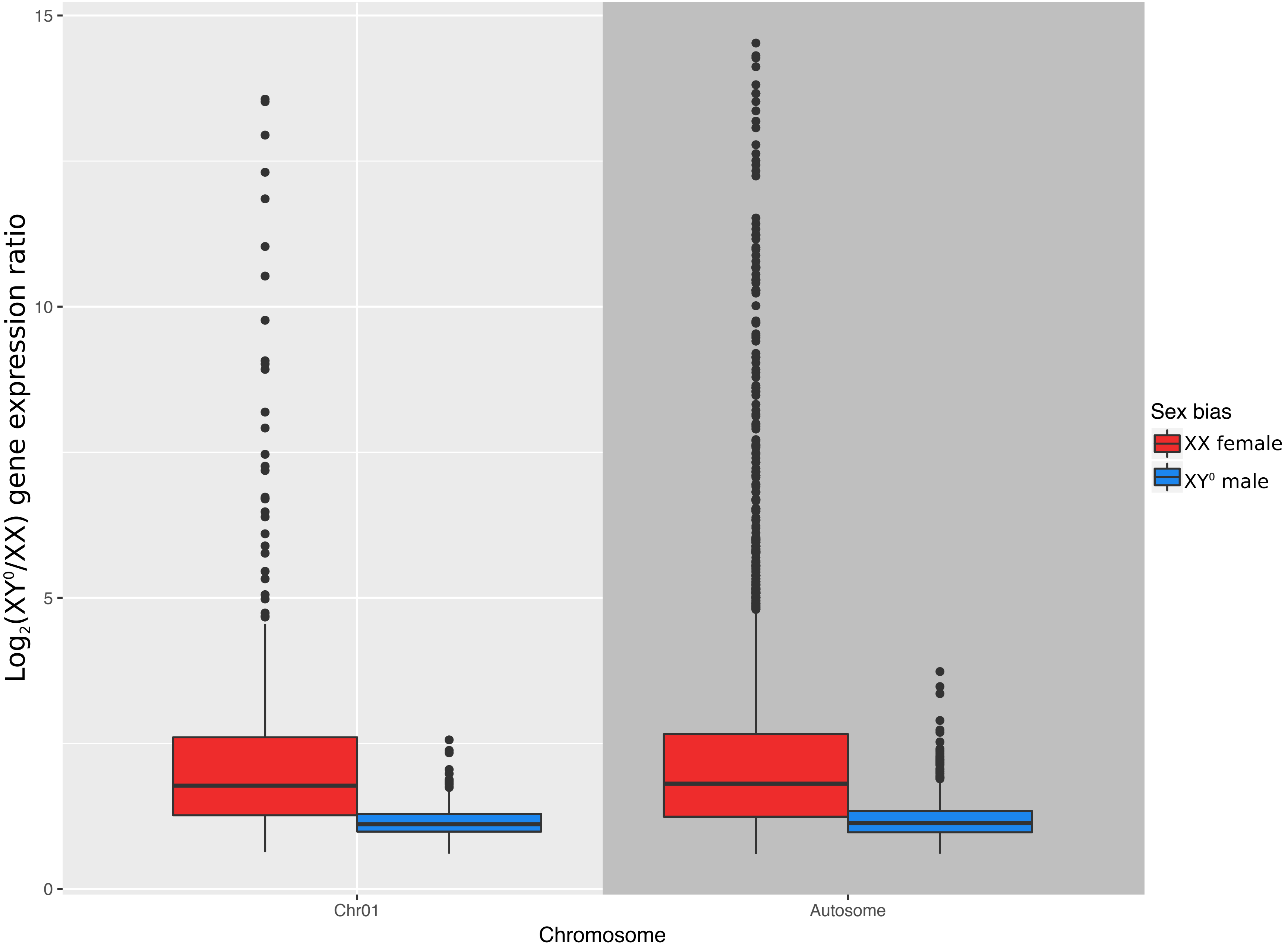

15
10
5
0
Log$_2$(XY^0/XX) gene expression ratio
Chr01
Autosome
Chromosome
Sex bias
XX female
XY^0 male

(c)

Figure S4. The genomic locations of sex-biased genes on both sex chromosome and autosomes at metamorph stage G43 (orthologs on *X. tropicalis* on autosomes are plotted on each chromosome [a], and orthologs on combined autosomes at stage G43 are plotted [b]), and at froglet stage G46 ([c], orthologs on autosomes are combined). Sex chromosome is on the left (Chr01), and autosomes are on the right in shade.

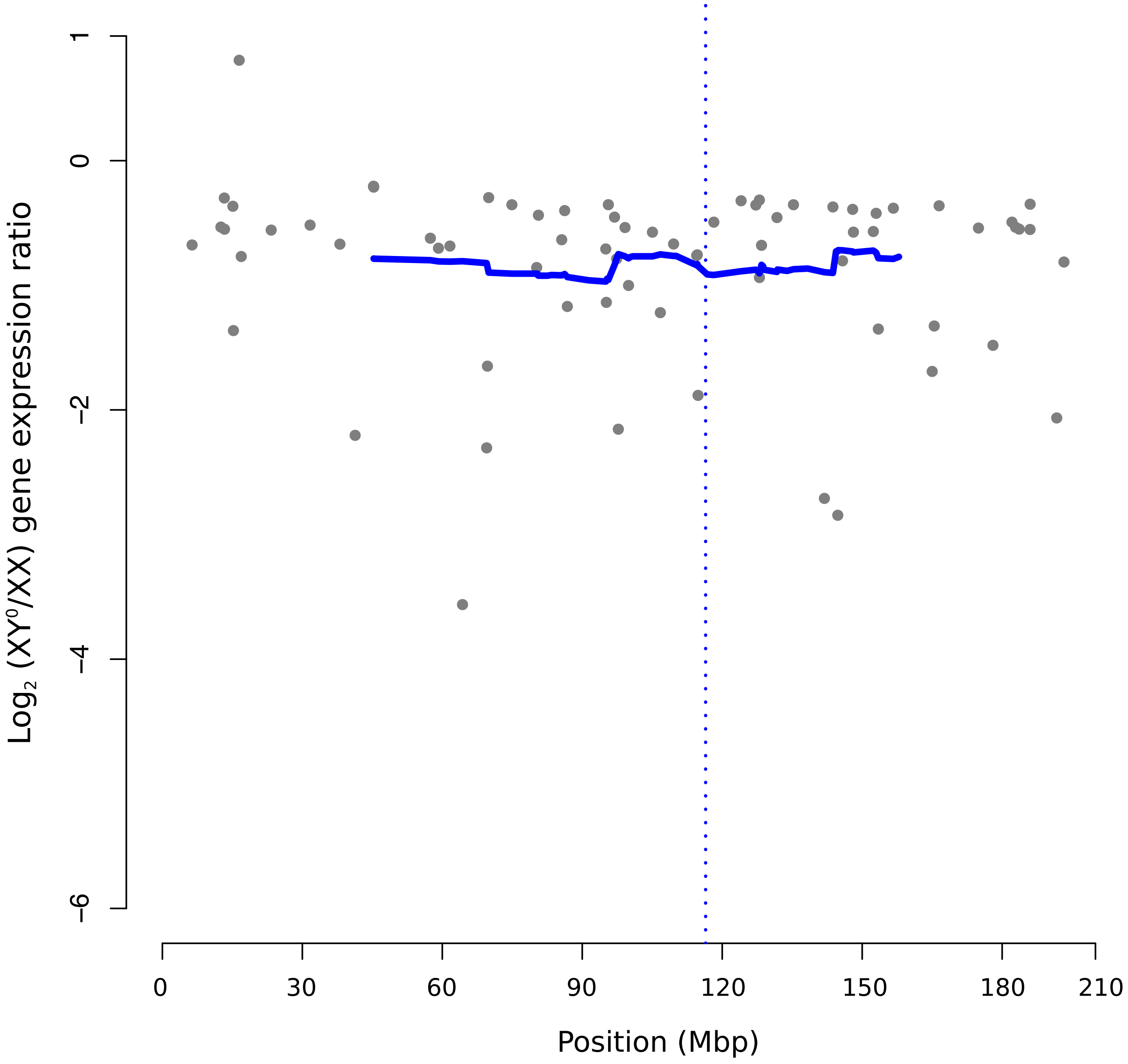
Log2 (XY0/XX) gene expression ratio
1
0
−2
−4
−6
0
30
60
90
120
150
180
210
Position (Mbp)

(a)

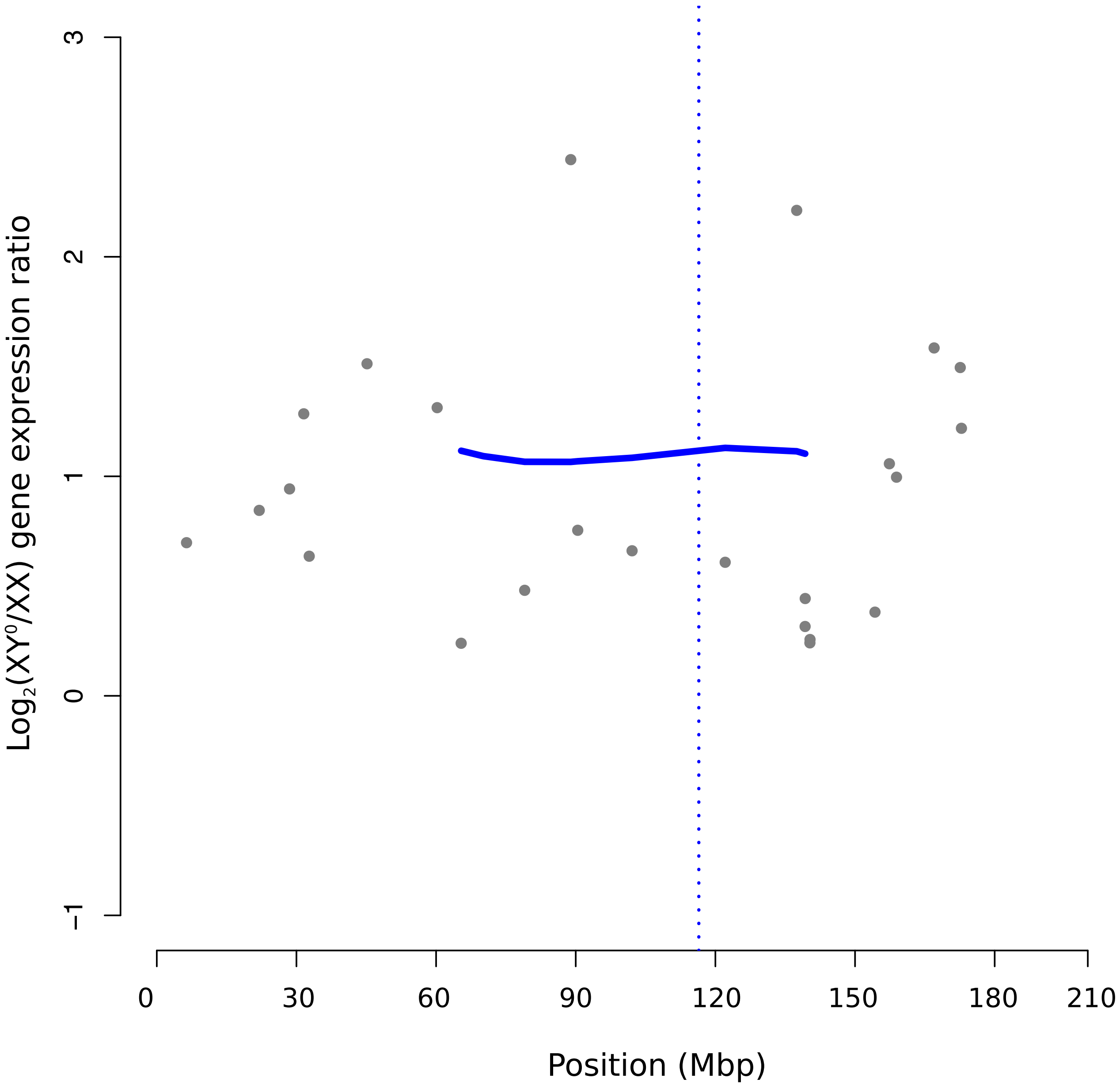

Log2(XY0/XX) gene expression ratio
3
2
1
0
−1
0
30
60
90
120
150
180
210
Position (Mbp)

(b)

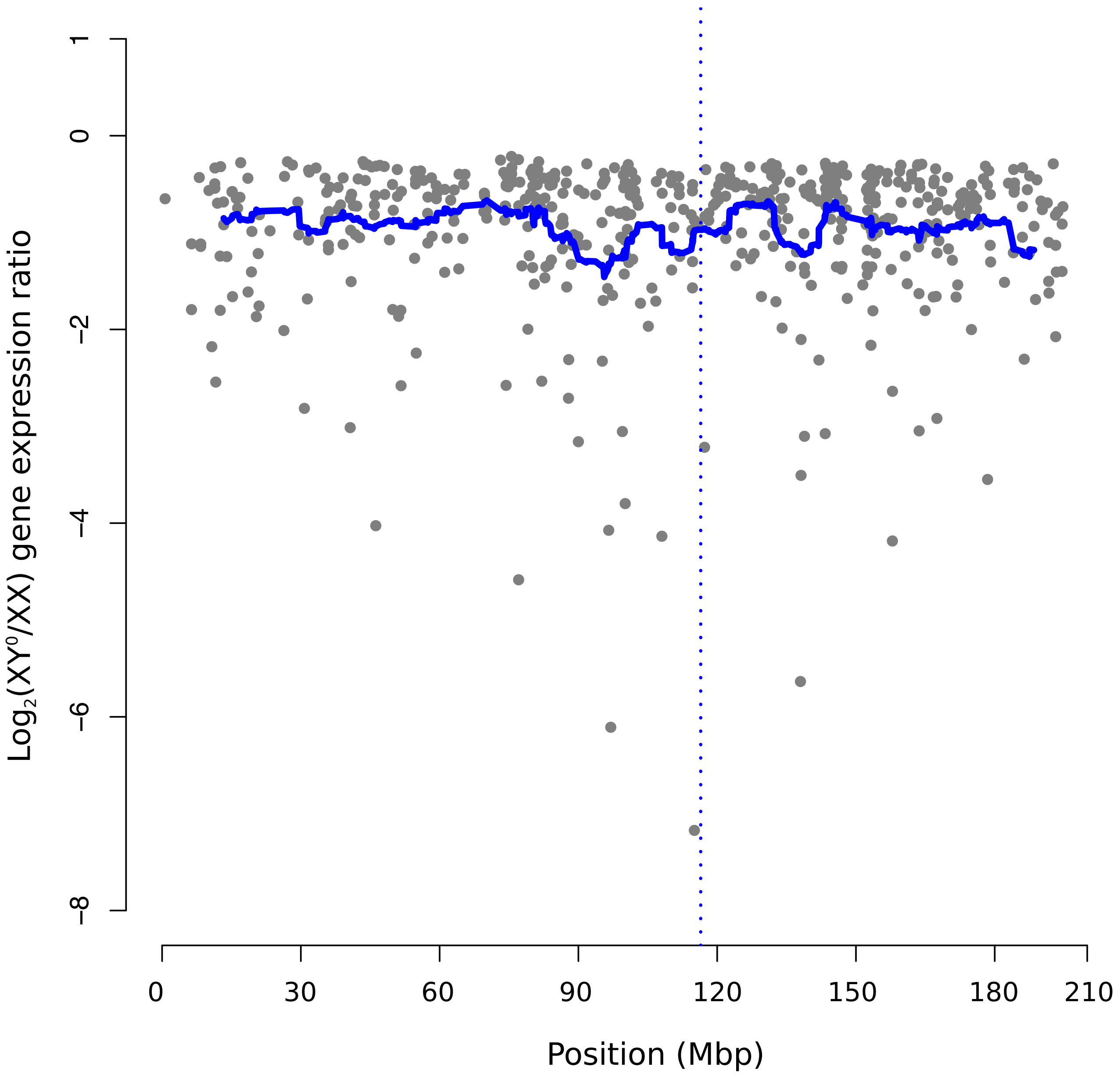

1
0
−2
−4
−6
−8
Log2(XY0/XX) gene expression ratio
0
30
60
90
120
150
180
210
Position (Mbp)

(c)

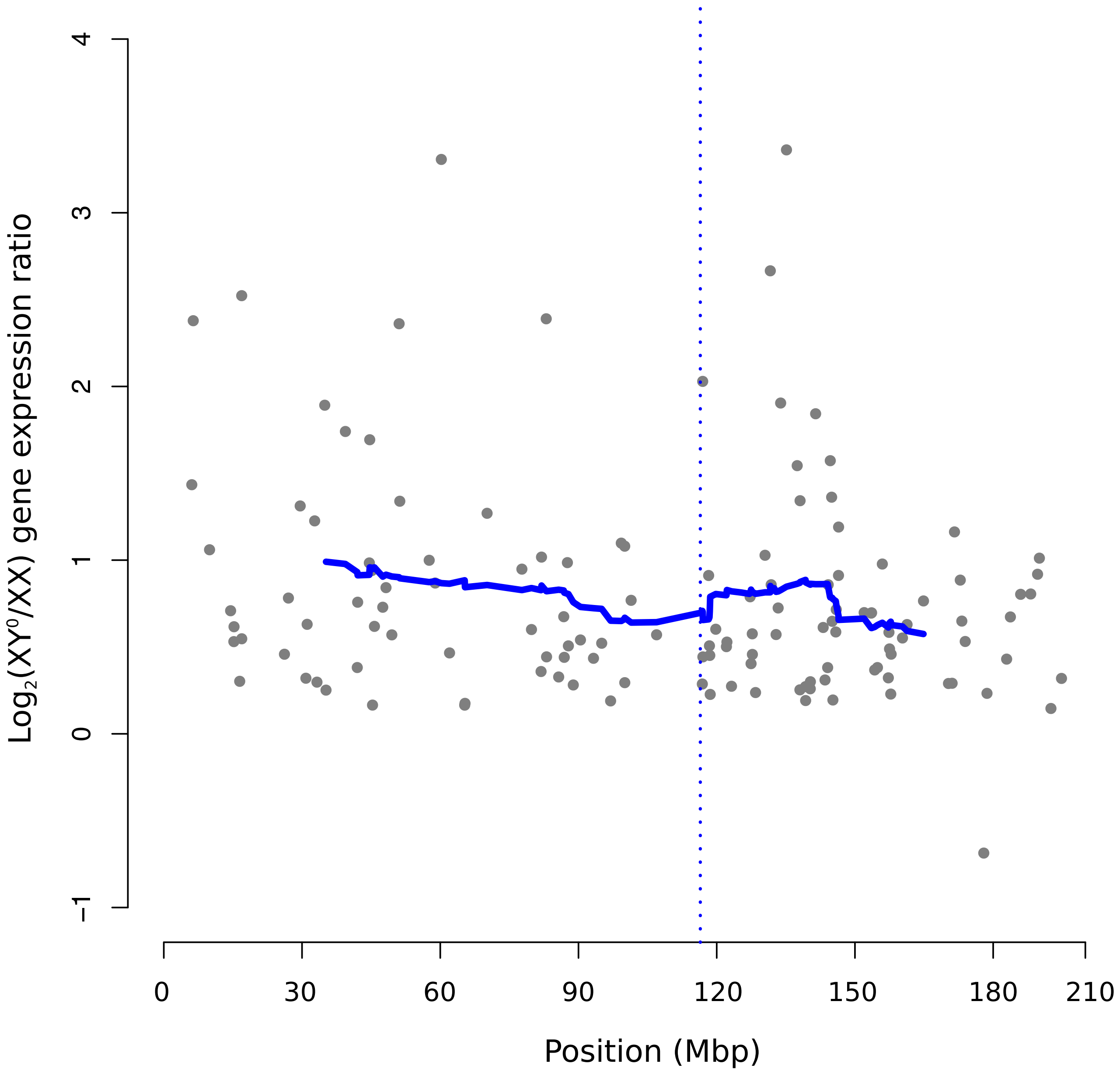

Log$_2$(XY^0/XX) gene expression ratio
4
3
2
1
0
−1
0
30
60
90
120
150
180
210
Position (Mbp)

(d)

Figure S5. Manhattan plot of $log_2(XY^0/XX)$ gene expression ratio for sex-biased genes along the sex chromosome: XX-biased (a) and XY^0-biased genes (b) on stage G43, and XX-biased (c) and XY^0-biased genes (d) on stage G46, with a sliding window of 20 genes for stage G43 and of 40 genes for stage G46.

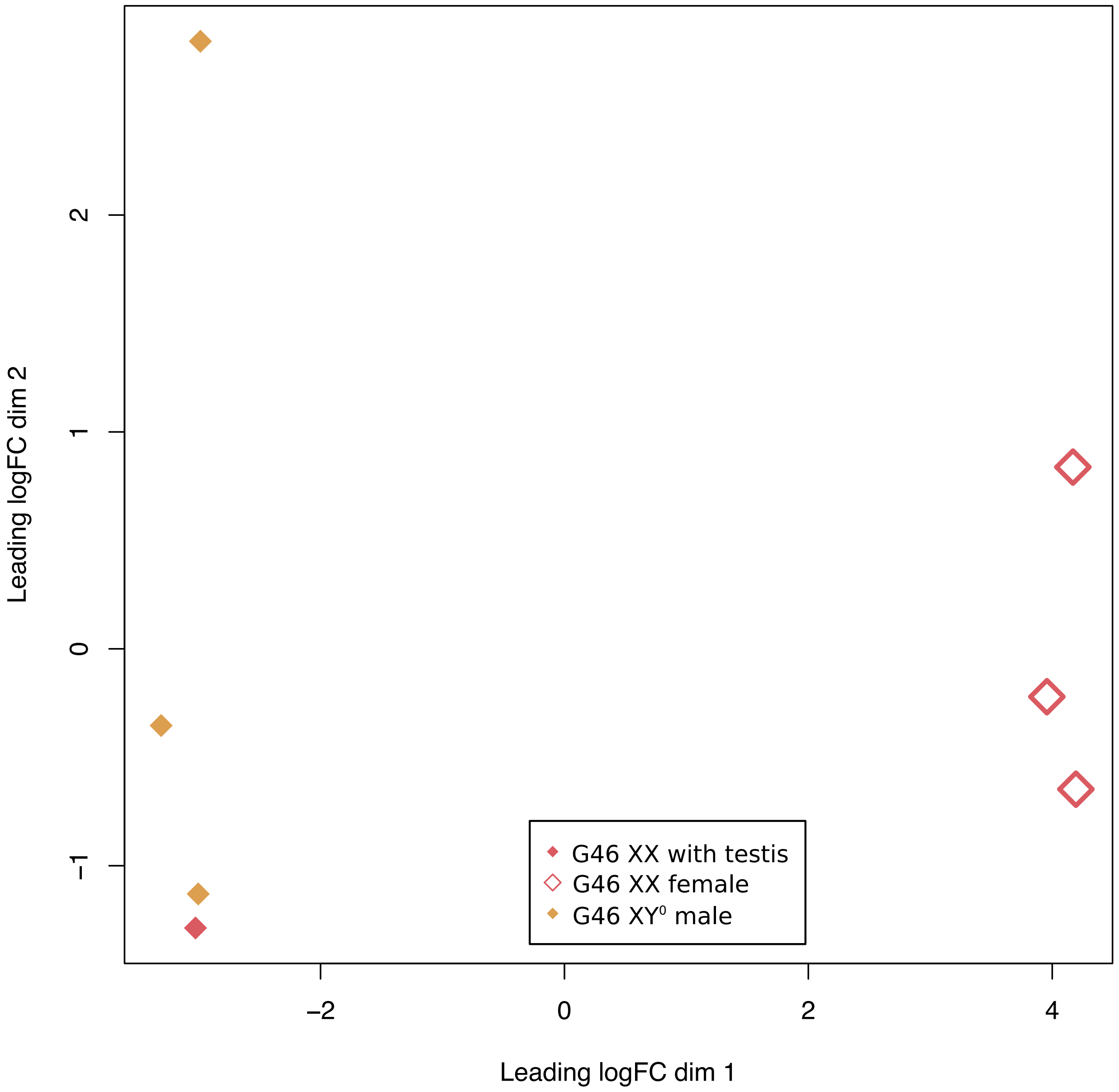

Figure S6. Multi-dimensional scaling (MDS) plot for total gene expression profile at stage G46.

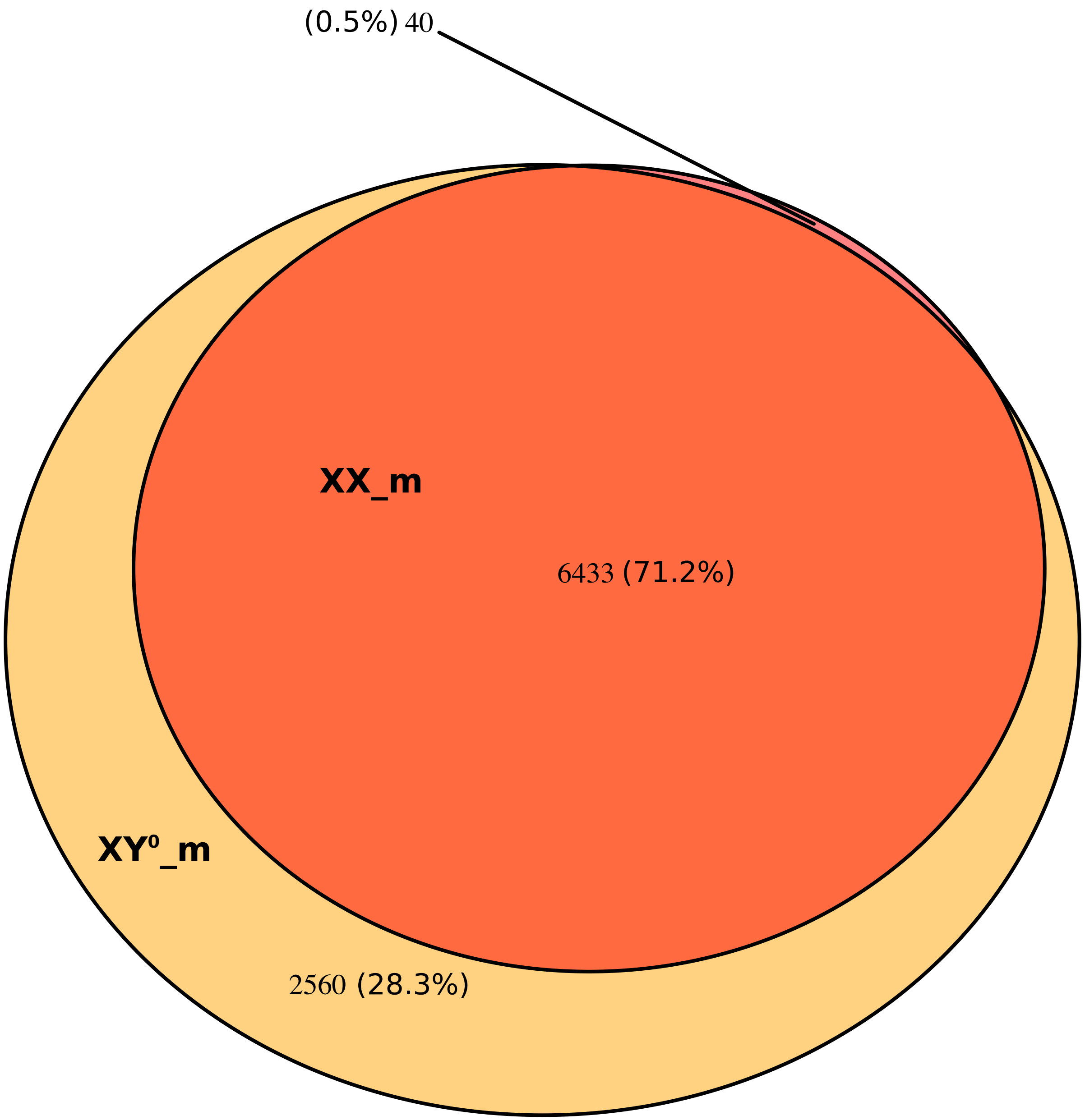

(a)

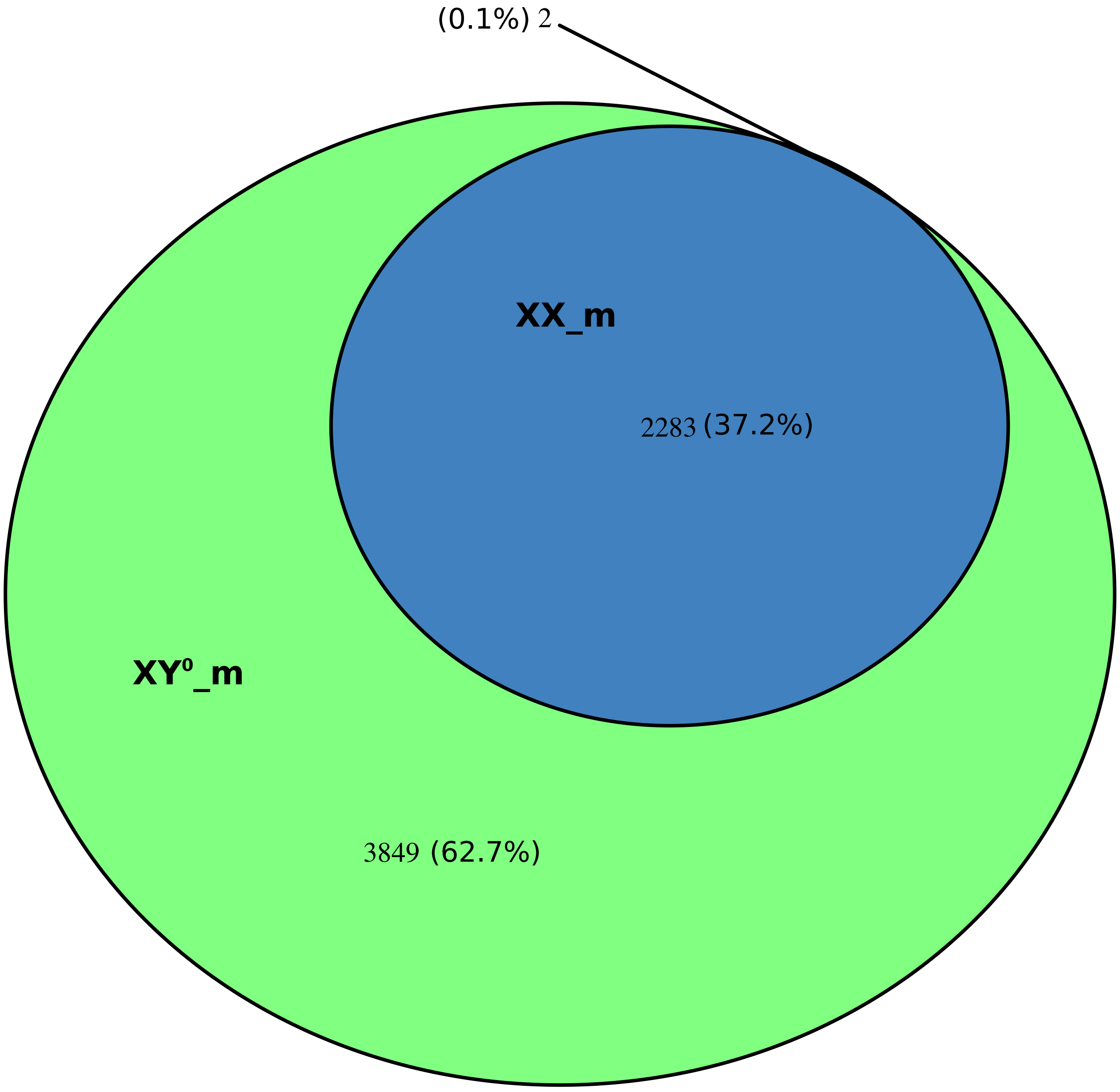

(b)

Figure S7. Venn diagram of identified female-biased genes, in contrast between XX females with XY° males (XY°_m, in yellow) or XX male (XX_m, in orange; a), and identified male-biased genes in contrast between XX females and XY^0 males (XY^0_m, in green) or with the XX male (XX_m, in blue; b) at stage G46. Differentially expressed genes with fold change ≥2, FDR < 0.05 are reported.

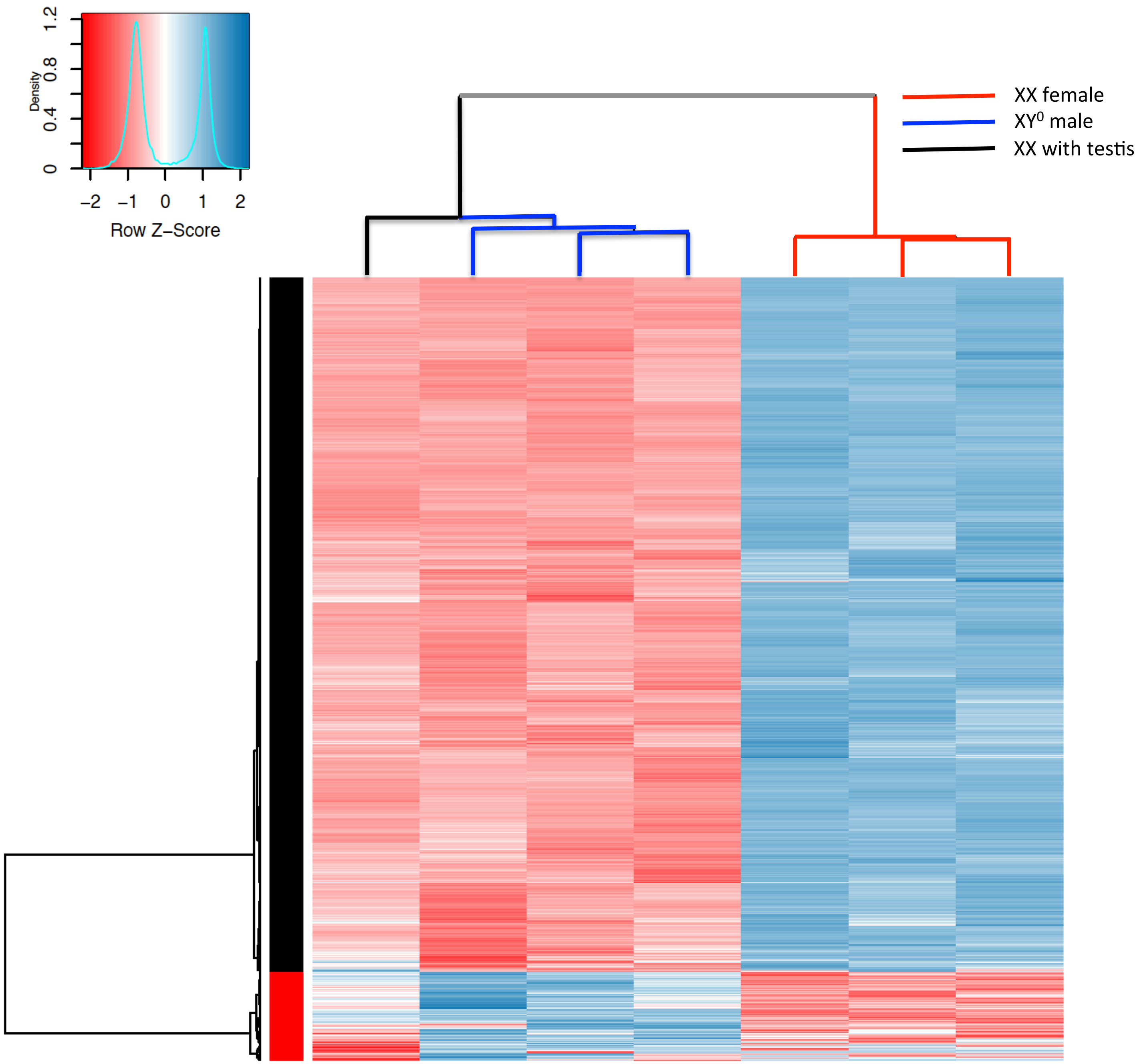

Density
0
0.4
0.8
1.2
−2
−1
0
1
2
Row Z−Score
XX female
XY0 male
XX with testis

Figure S8. Heatmaps and hierarchical clustering of differentially expressed genes (FDR < 0.05) based on genotypic sex, between XX (including XX male and XX females) and XY^0 (male) individuals. Blue and red colors represent high and low expression, respectively.

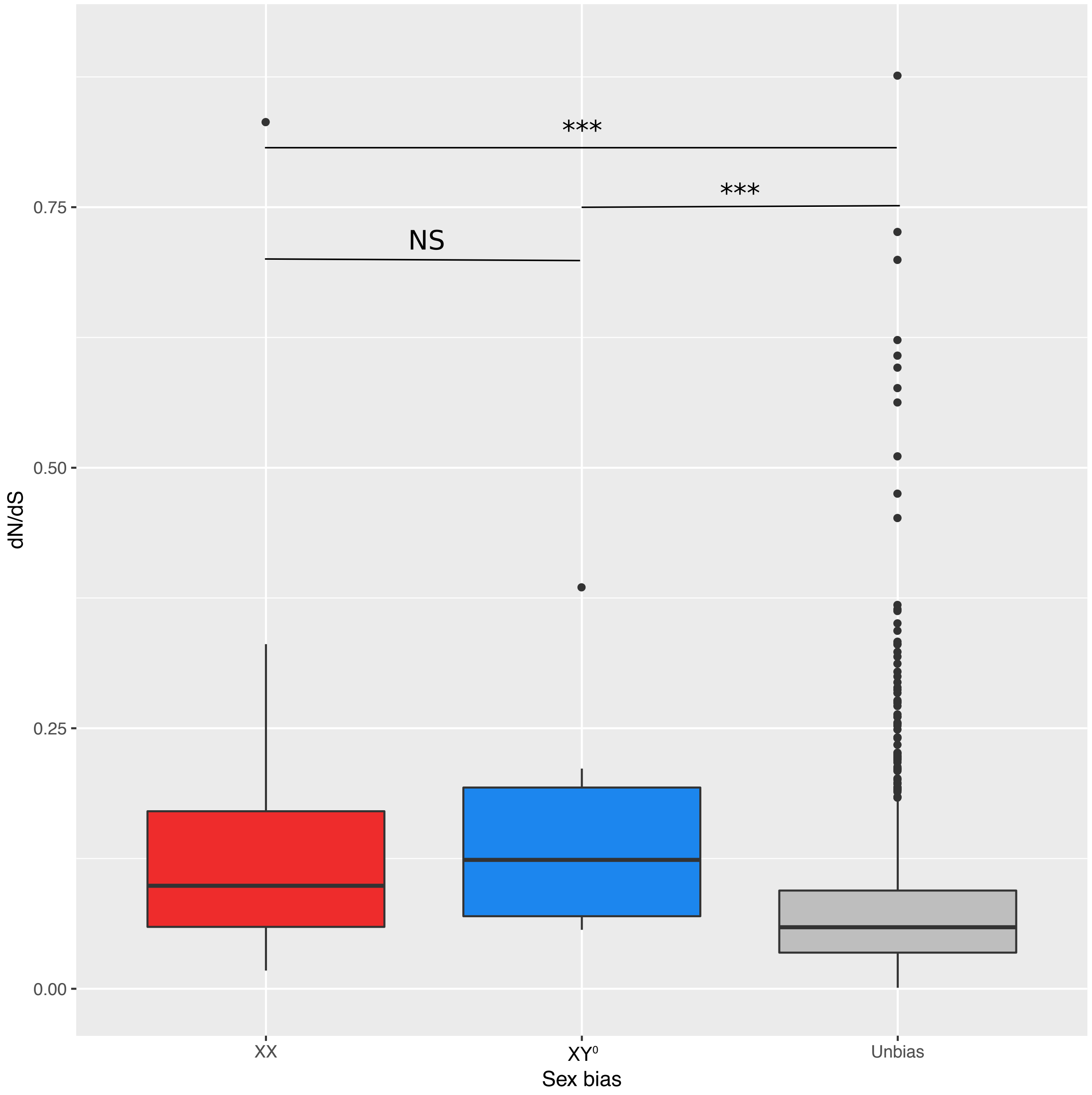

(a)

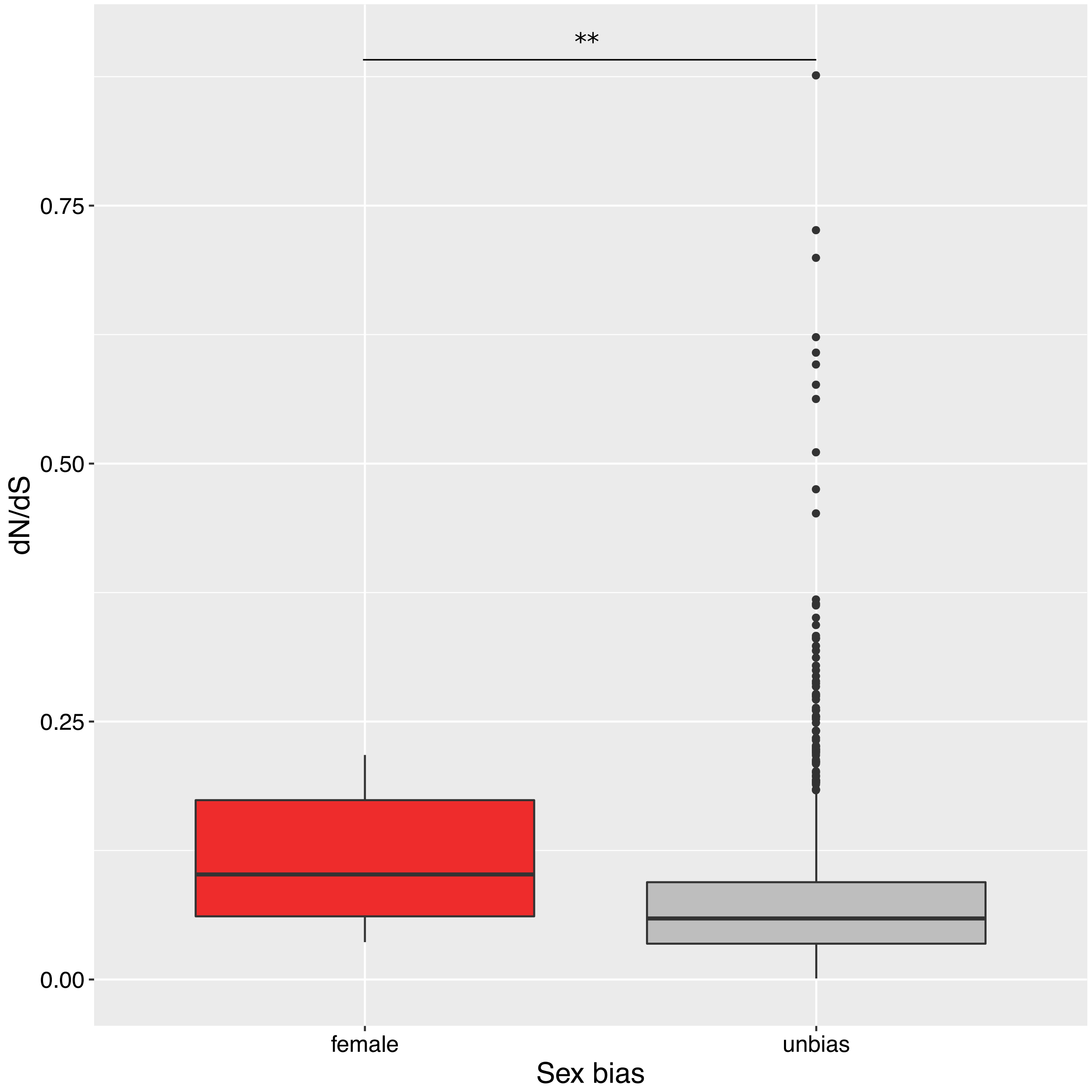

(b)

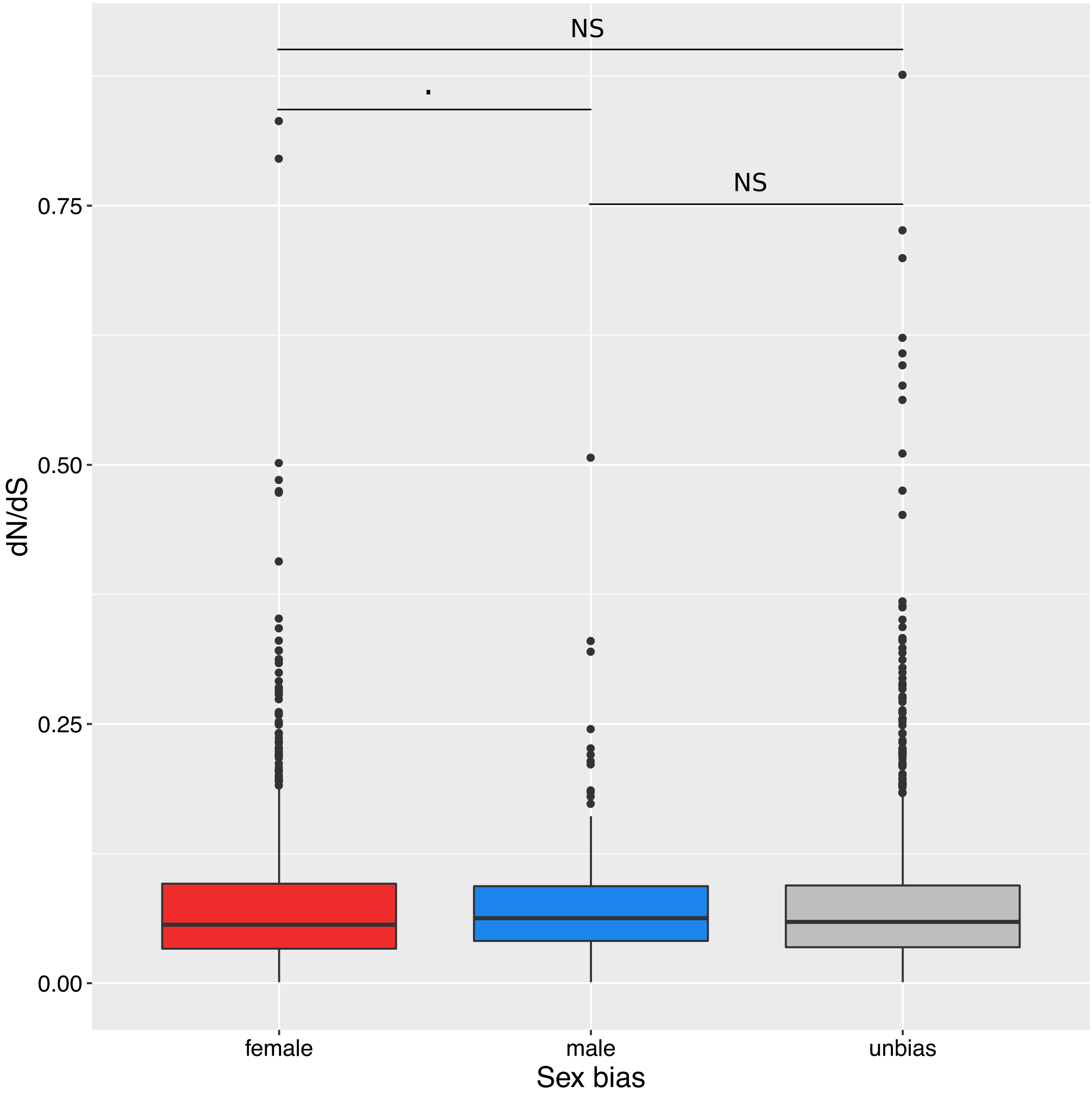

(c)

Figure S9. Ratios of nonsynonymous to synonymous substitutions *dN/dS* for shared sex-biased genes (XX- and XY°- biased) between the two stages G43 and G46 (a), *dN/dS* for XY°-female-biased genes and unbiased genes resulting from differentially gene expression analysis (FDR < 0.05, Log2FC >1) between XY° females and XY^0 males at stage G43 (b), and *dN/dS* for XX-female-biased, XX-male-biased and unbiased genes from differentially gene expression analysis between XX females and XX male at stage G46 (c). Significance is indicated by asterisks: *** codes are, 0.001 '***', 0.01 '**', 0.05 '*', 0.1 '.', not significant 'NS'.

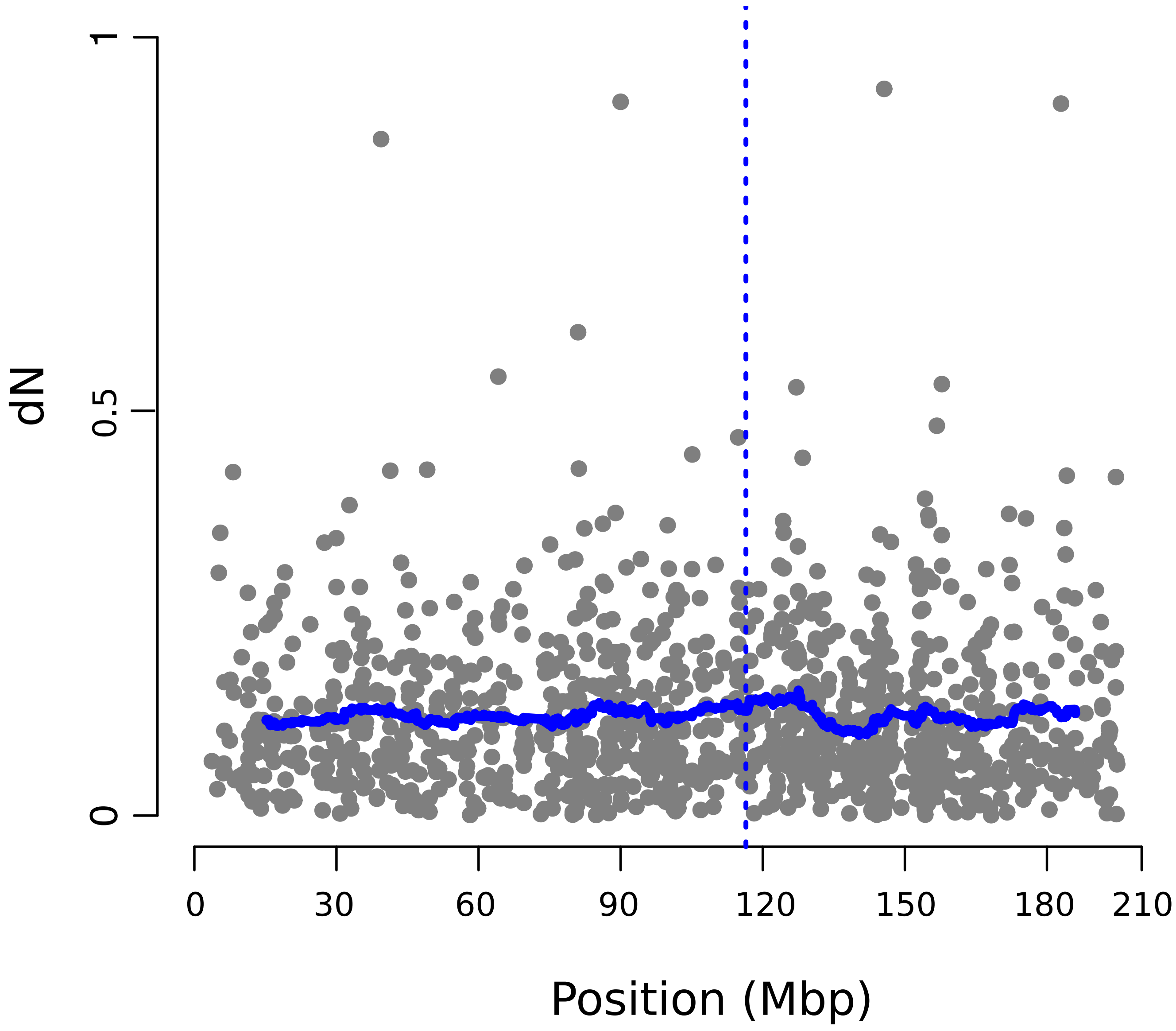

(a)

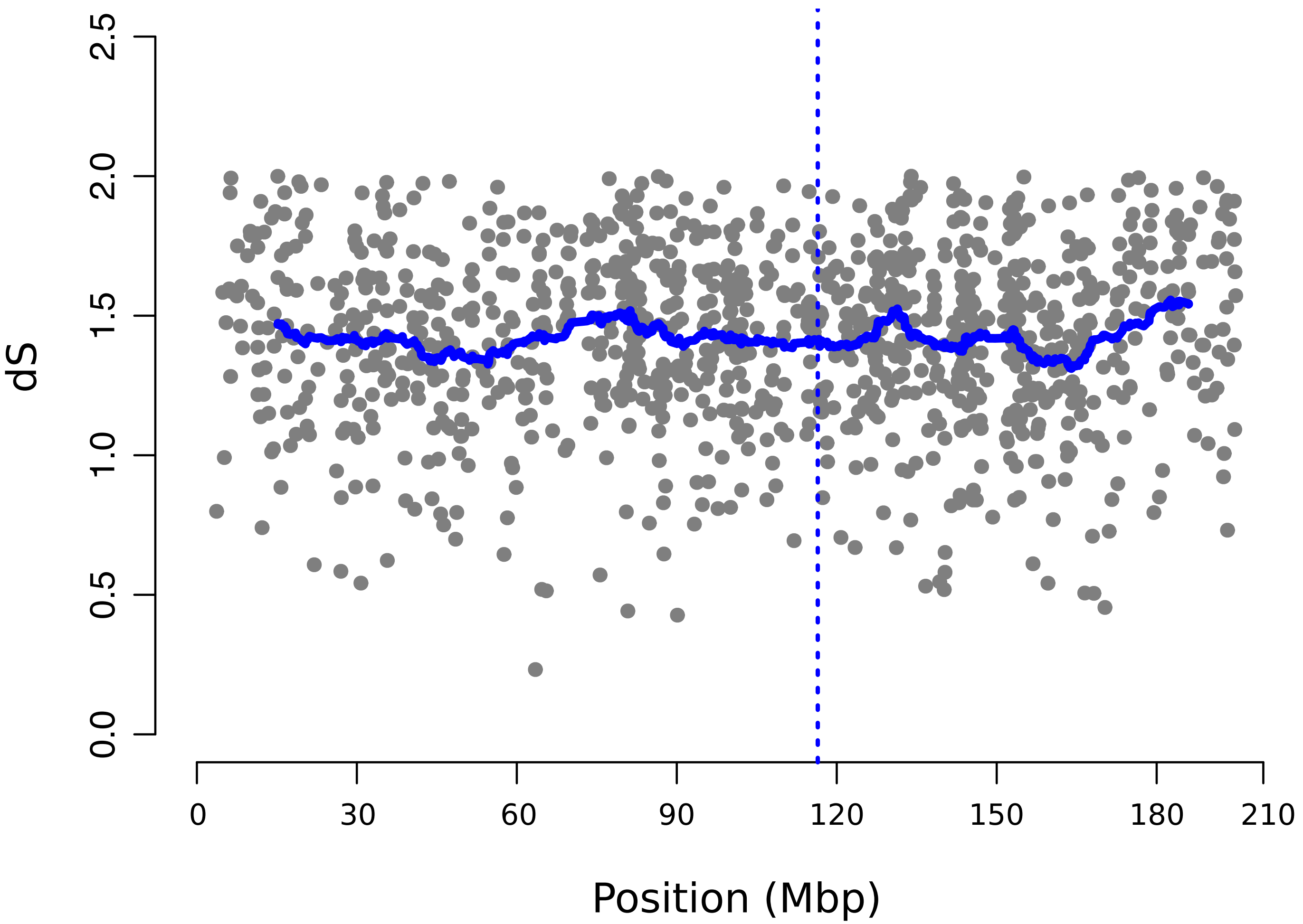

(b)

Figure S10. Nonsynonymous substitutions (d_N) (a) and synonymous substitutions (d_S) (b) for genes along the sex chromosome, with a sliding window (non-overlapping) of 40 genes. Both show no special pattern around the sex-determining region. The horizontal blue line shows the average value of a sliding window of 40 genes, and *Dmrt1* position is marked by the vertical blue dotted line.

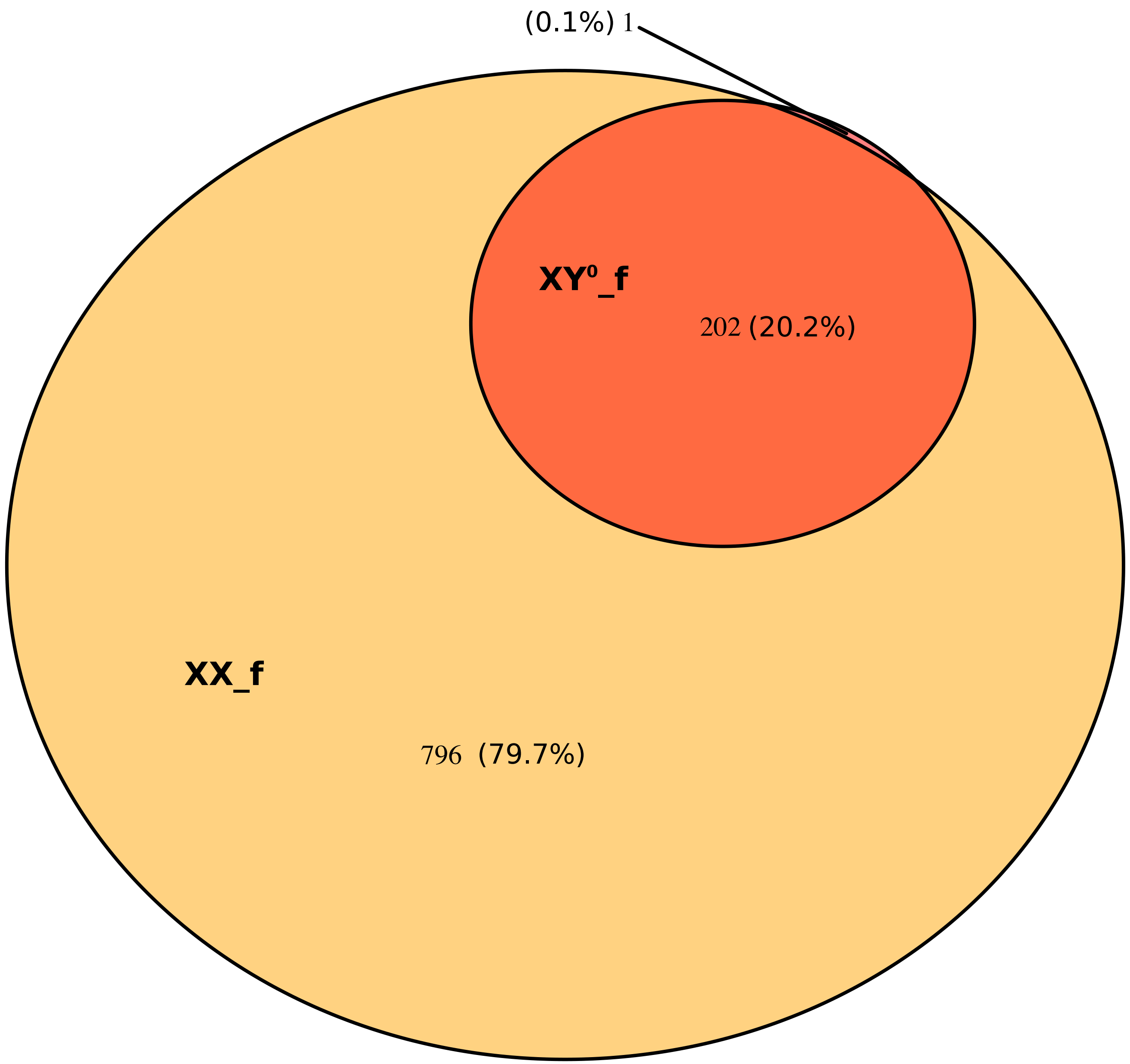
(0.1%) 1
XY⁰_f
202 (20.2%)
XX_f
796 (79.7%)

Figure S11. Venn diagrams of shared female-biased genes in contrast between XY° males and XY° females (XY°_f, in orange) or XX females (XX_f, in yellow) at stage G43.

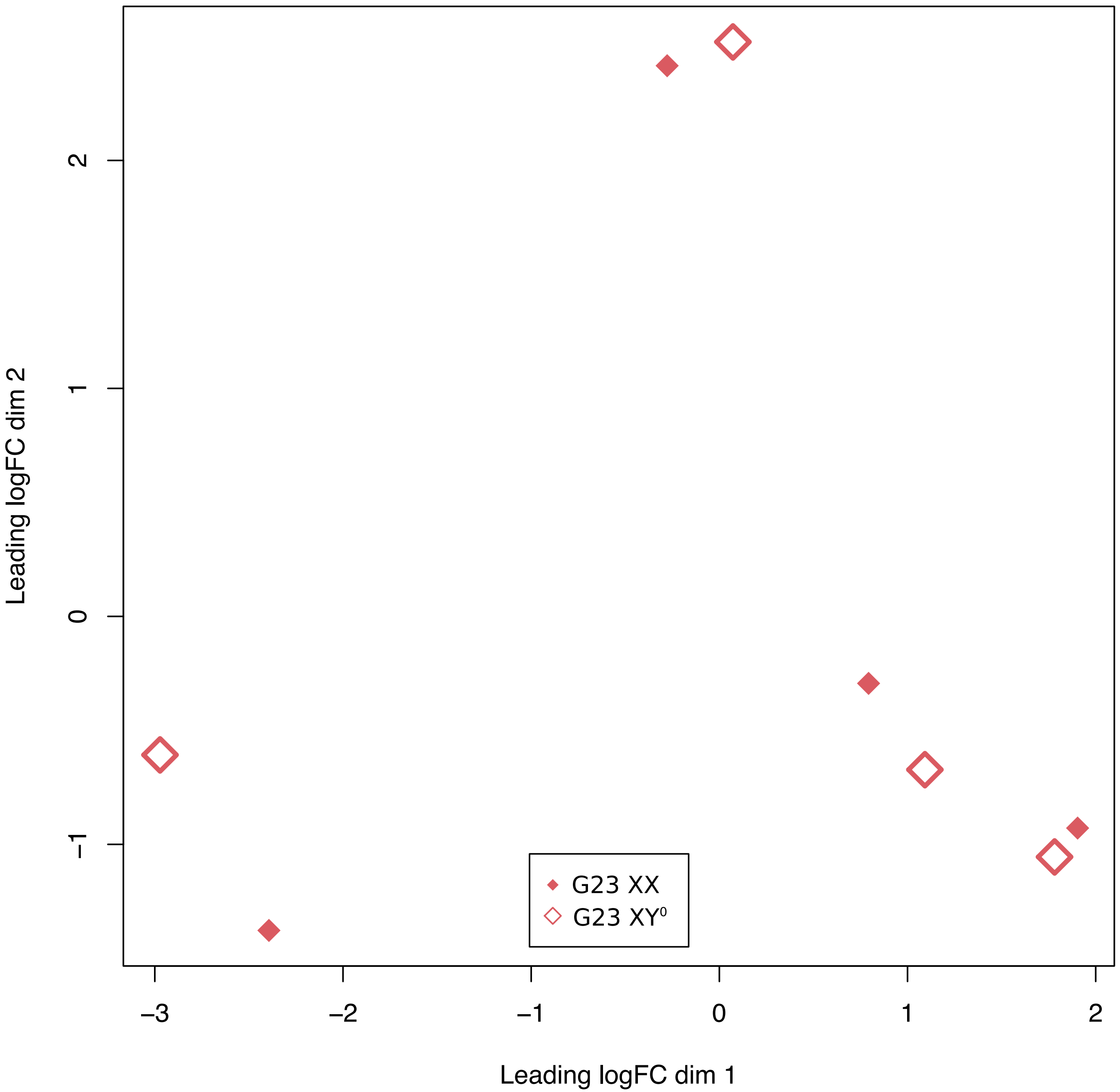
Leading logFC dim 2
2
1
0
−1
−3
−2
−1
0
1
2
Leading logFC dim 1
G23 XX
G23 XY0

(a)

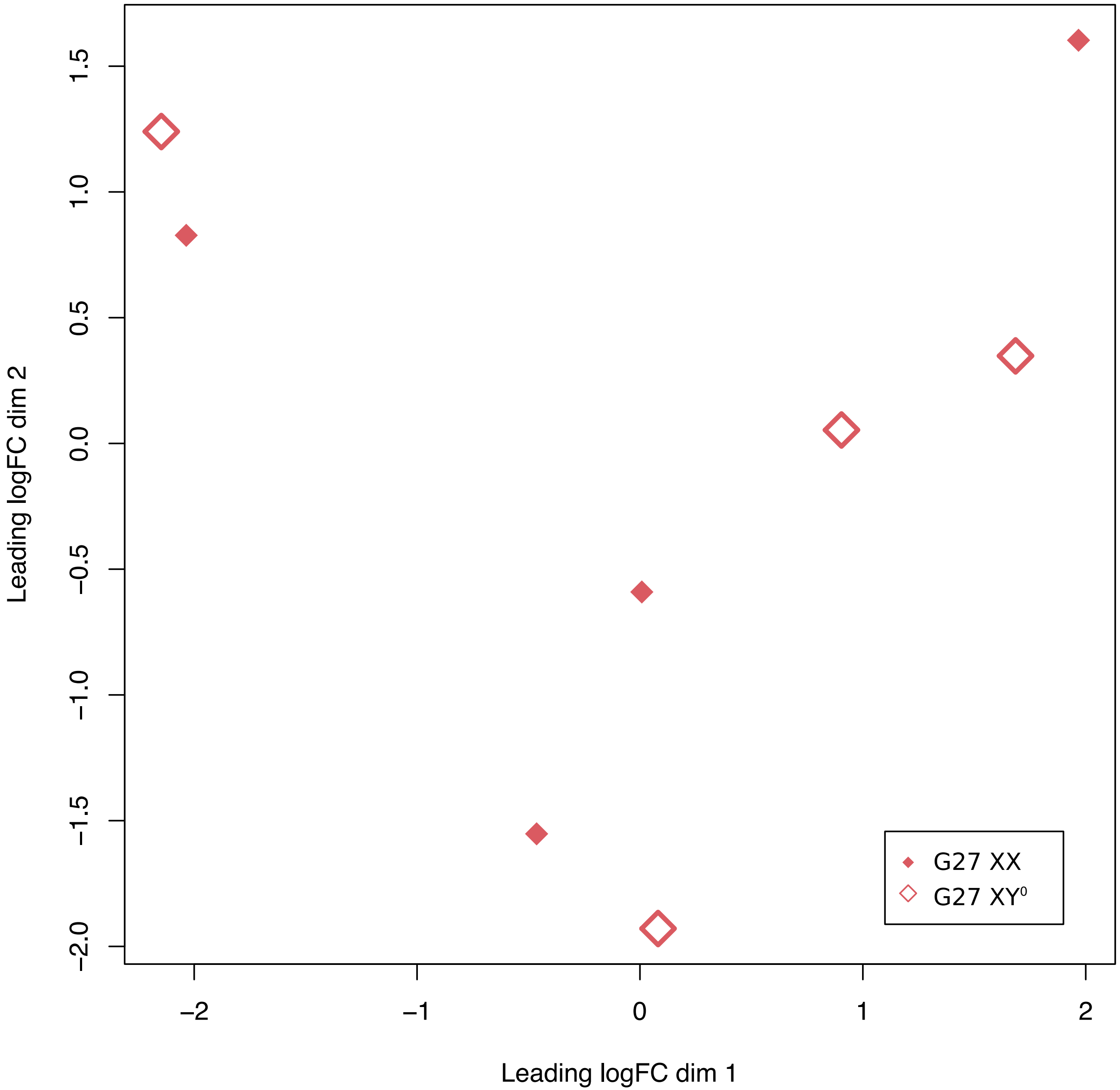

1.5
1.0
0.5
0.0
−0.5
−1.0
−1.5
−2.0
Leading logFC dim 2
−2
−1
0
1
2
Leading logFC dim 1
G27 XX
G27 XY0

(b)

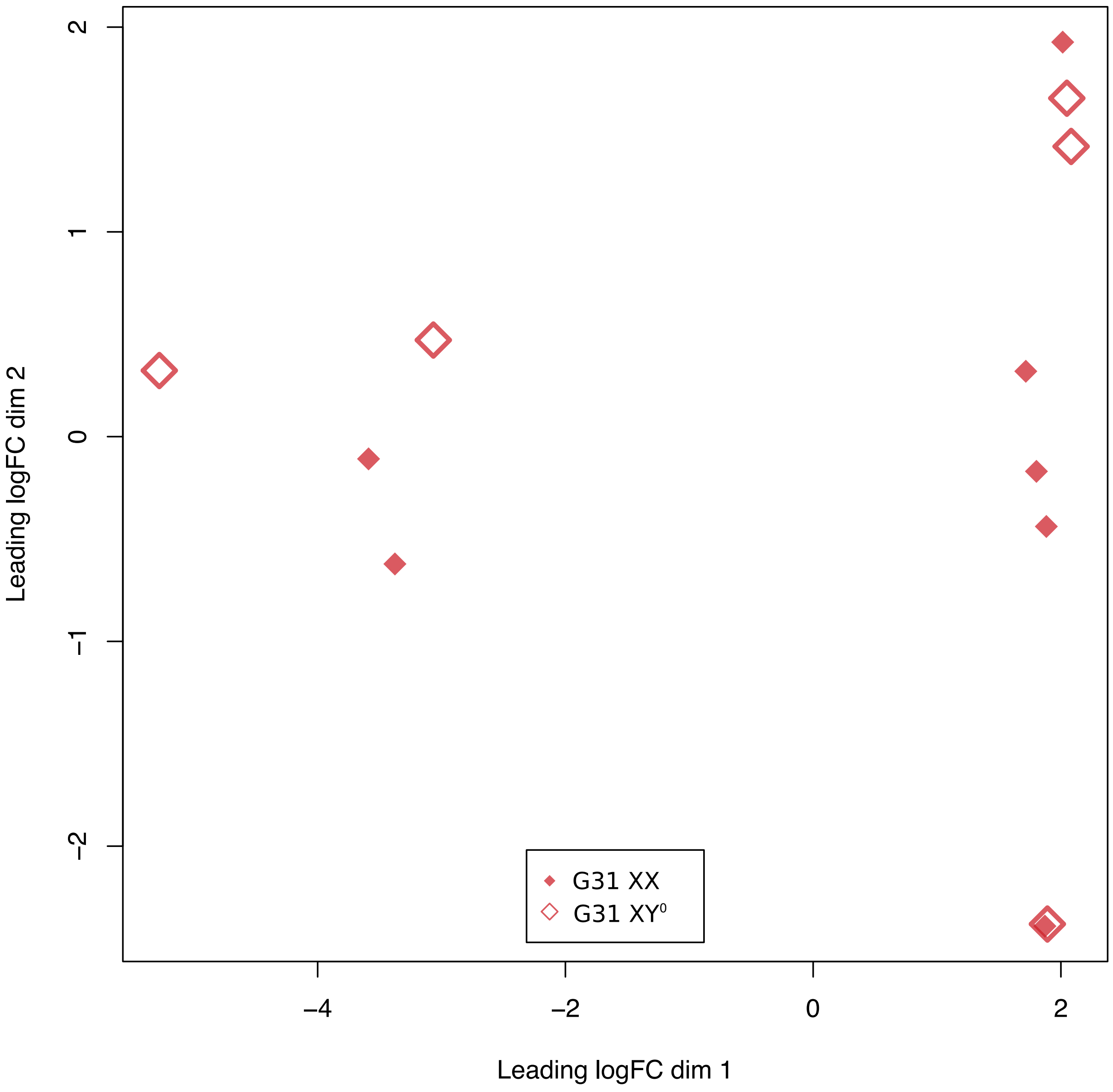

Leading logFC dim 2
Leading logFC dim 1
2
1
0
−1
−2
−4
−2
0
2
G31 XX
G31 XY⁰

(c)

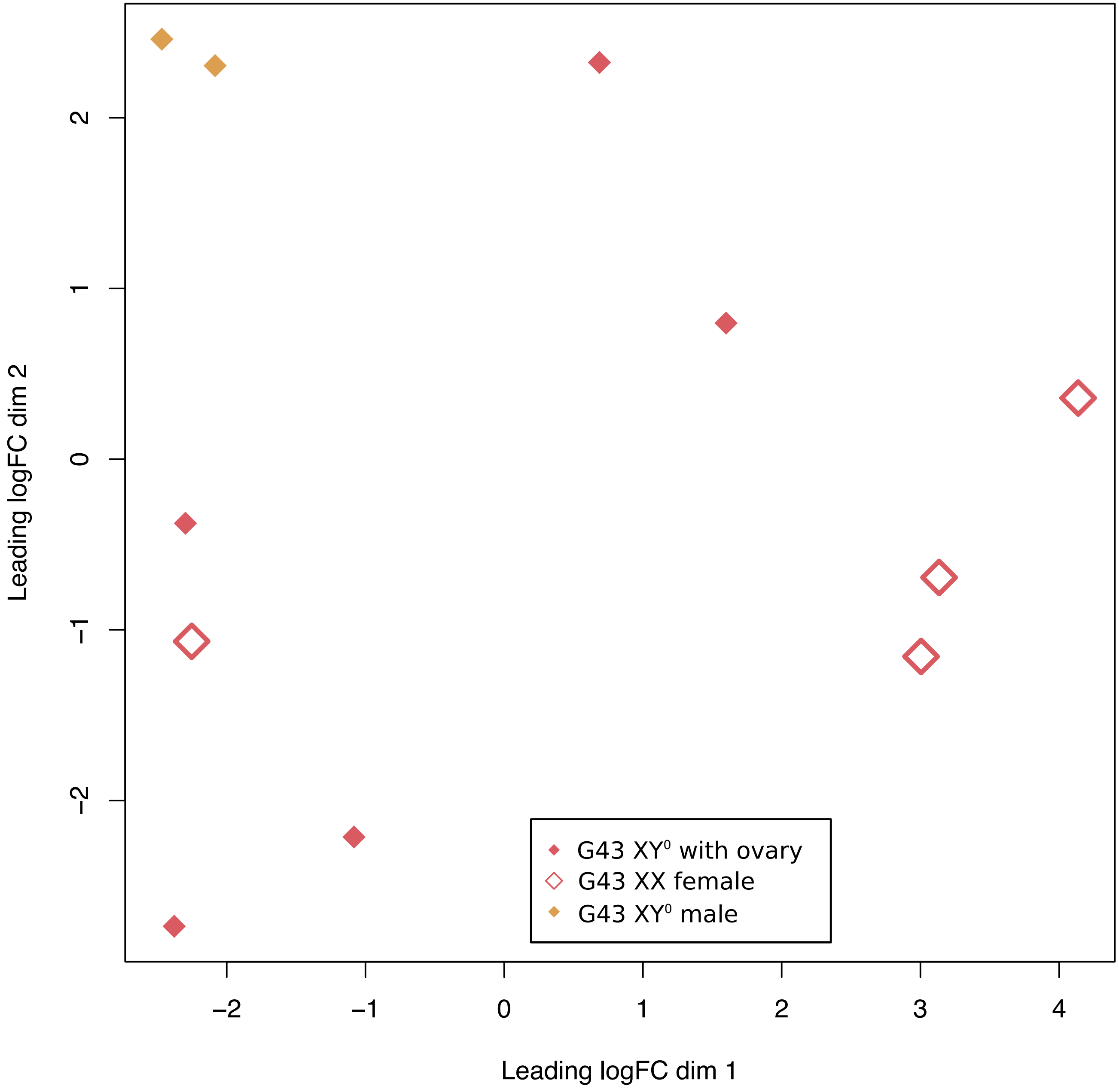
Leading logFC dim 2
Leading logFC dim 1
2
1
0
−1
−2
−2
−1
0
1
2
3
4
G43 XY⁰ with ovary
G43 XX female
G43 XY⁰ male

(d)

Figure S12. Multi-dimensional scaling (MDS) plots for total gene expression profile at four developmental stages: G23 (a), G27 (b), G31 (c), G43 (d).
