# Supplementary material for: Evolutionary and developmental dynamics of sex-biased gene expression in common frogs with proto-Y chromosomes
Source: Genome Biol. 2018 Oct 5;19:156. doi: 10.1186/s13059-018-1548-4 (PMC6173898; doi:10.1186/s13059-018-1548-4)
Supplement: Supplementary file 4 — Table S1. Genomic location distributions of one-to-one ortholog of reciprocal best BLAST hit between R. temporaria and X. tropicalis. *Ratio between R. temporaria ortholog number and the number of X. tropicalis gene number per chromosome. Table S2. GO enrichment analysis for differentially expressed gene clustering groups at stage G43. GO depicts three complementary biological concepts including biological process (BP), molecular function (MF), and cellular component (CC). (DOC 98 kb) [file 13059_2018_1548_MOESM4_ESM.doc]

**Table S1**. Genomic location distributions of one-to-one ortholog of reciprocal best blast hit between *R. temporaria* and *X. tropicalis*. * ratio between *R. temporaria* ortholog number and the number of *X. tropicalis* gene number per chromosome.

| ***X. tropicalis* Chr.** | **Gene number** | ***R. temporaria* ortholog number** | **Ratio*** | **Chisq test** |
| --- | --- | --- | --- | --- |
| **1** | 3196 | 1468 | 0.46 | NA |
| **2** | 2646 | 1310 | 0.5 | NA |
| **3** | 2639 | 1186 | 0.45 | NA |
| **4** | 2325 | 1175 | 0.51 | NA |
| **5** | 2074 | 955 | 0.46 | NA |
| **6** | 1645 | 779 | 0.47 | NA |
| **7** | 1940 | 848 | 0.44 | NA |
| **8** | 2436 | 1038 | 0.43 | NA |
| **9** | 1785 | 613 | 0.34 | P < 0.0001 |
| **10** | 948 | 482 | 0.51 | NA |

**Table S2**: GO enrichment analysis for differentially expressed gene clustering groups at stage G43. GO depicts three complementary biological concepts including Biological Process (BP), Molecular Function (MF), and Cellular Component (CC).

| **Set 1** | GO characterization | Fisher-test P value |
| --- | --- | --- |
| BP |  |  |
| GO:2000360 | negative regulation of binding of sperm ... | 1.70E-19 |
| GO:2000344 | positive regulation of acrosome reaction | 1.70E-19 |
| GO:0060478 | acrosomal vesicle exocytosis | 1.70E-19 |
| GO:0002922 | positive regulation of humoral immune re... | 4.70E-17 |
| GO:0070528 | protein kinase C signaling | 1.30E-15 |
| GO:0042102 | positive regulation of T cell proliferat... | 2.80E-14 |
| GO:0045860 | positive regulation of protein kinase ac... | 1.30E-08 |
| GO:0048599 | oocyte development | 3.00E-12 |
| GO:0010737 | protein kinase A signaling | 5.50E-09 |
| GO:0007338 | single fertilization | 6.80E-28 |
| GO:2000196 | positive regulation of female gonad deve... | 1.80E-07 |
| GO:0045956 | positive regulation of calcium ion-depen... | 1.80E-07 |
| GO:0071073 | positive regulation of phospholipid bios... | 1.80E-07 |
| GO:0001807 | regulation of type IV hypersensitivity | 1.80E-07 |
| GO:0002885 | positive regulation of hypersensitivity | 1.80E-07 |
| GO:0001547 | antral ovarian follicle growth | 5.30E-07 |
| GO:0032753 | positive regulation of interleukin-4 pro... | 1.20E-06 |
| GO:0032729 | positive regulation of interferon-gamma ... | 1.20E-06 |
| GO:0001825 | blastocyst formation | 2.40E-06 |
| GO:0007339 | binding of sperm to zona pellucida | 4.10E-24 |
| GO:0043046 | DNA methylation involved in gamete gener... | 2.60E-05 |
| GO:0048015 | phosphatidylinositol-mediated signaling | 5.30E-05 |
| GO:0051897 | positive regulation of protein kinase B ... | 7.50E-05 |
| GO:0045143 | homologous chromosome segregation | 9.00E-05 |
| GO:0002711 | positive regulation of T cell mediated i... | 9.60E-05 |
| GO:0090280 | positive regulation of calcium ion impor... | 0.00012 |
| GO:0048639 | positive regulation of developmental gro... | 0.00012 |
| GO:0002455 | humoral immune response mediated by circ... | 0.00015 |
| GO:0060467 | negative regulation of fertilization | 2.50E-23 |
| GO:0002687 | positive regulation of leukocyte migrati... | 0.00031 |
| GO:0006661 | phosphatidylinositol biosynthetic proces... | 0.00031 |
| GO:0000041 | transition metal ion transport | 0.00122 |
| GO:0007275 | multicellular organism development | 0.05158 |
| GO:0051092 | positive regulation of NF-kappaB transcr... | 0.00296 |
| GO:0048477 | oogenesis | 5.20E-14 |
| GO:0007067 | mitotic nuclear division | 0.00094 |
| GO:0007126 | meiotic nuclear division | 2.30E-06 |
| GO:0032897 | negative regulation of viral transcripti... | 0.00552 |
| GO:0006886 | intracellular protein transport | 0.08818 |
| GO:0007052 | mitotic spindle organization | 0.00818 |
| CC |  |  |
| GO:0005578 | proteinaceous extracellular matrix | 5.10E-14 |
| GO:0030141 | secretory granule | 6.30E-10 |
| GO:0005794 | Golgi apparatus | 0.00042 |
| GO:0005771 | multivesicular body | 1.10E-07 |
| GO:0002080 | acrosomal membrane | 1.60E-06 |
| GO:0005886 | plasma membrane | 0.00315 |
| MF |  |  |
| GO:0005384 | manganese ion transmembrane transporter ... | 1.30E-07 |
| GO:0004869 | cysteine-type endopeptidase inhibitor ac... | 0.00026 |
| GO:0004871 | signal transducer activity | 0.00078 |
|  |  |  |
| **Set 2** |  |  |
| BP |  |  |
| GO:0032197 | transposition, RNA-mediated | 0.00025 |
| GO:0042572 | retinol metabolic process | 0.02093 |
| CC |  |  |
| GO:0010494 | cytoplasmic stress granule | 0.00012 |
| GO:0034361 | very-low-density lipoprotein particle | 0.00604 |
| MF |  |  |
| GO:0003727 | single-stranded RNA binding | 0.0027 |
| GO:0003697 | single-stranded DNA binding | 0.0035 |
|  |  |  |
| **Set 3** |  |  |
| BP |  |  |
| GO:0006414 | translational elongation | 0.00116 |
| GO:0030855 | epithelial cell differentiation | 0.00136 |
| GO:0006412 | translation | 0.00016 |
| CC |  |  |
| GO:0022627 | cytosolic small ribosomal subunit | 6.10E-05 |
| GO:0016324 | apical plasma membrane | 0.011 |
| MF |  |  |
| GO:0003735 | structural constituent of ribosome | 3.50E-05 |
| GO:0031369 | translation initiation factor binding | 0.018 |
|  |  |  |
| **Set 4** |  |  |
| BP |  |  |
| GO:0035195 | gene silencing by miRNA | 0.0054 |
| GO:0042220 | response to cocaine | 0.0054 |
| GO:0097028 | dendritic cell differentiation | 0.0063 |
| MF |  |  |
| GO:0005525 | GTP binding | 0.004 |
| GO:0016936 | galactoside binding | 0.013 |
